# Global infectious disease risks associated with occupational exposure among non-healthcare workers: a systematic review of the literature

**DOI:** 10.1136/oemed-2020-107164

**Published:** 2021-05-25

**Authors:** Sofie Acke, Simon Couvreur, Wichor M Bramer, Marie-Noëlle Schmickler, Antoon De Schryver, Juanita A Haagsma

**Affiliations:** 1 Family Medicine and Population Health (FAMPOP), Faculty of Medicine and Health Sciences, University of Antwerp, Wilrijk, Belgium; 2 Research and Development, Mensura Occupational Health Services, Brussel, Belgium; 3 Department of Twin Research, King's College London, London, UK; 4 Medical Library, Erasmus MC, Rotterdam, The Netherlands; 5 Department of Public Health, Erasmus MC, Rotterdam, Zuid-Holland, The Netherlands

**Keywords:** communicable diseases, military personnel, zoonoses, respiratory system, occupational health

## Abstract

**Objectives:**

Employees in non-healthcare occupations may be in several ways exposed to infectious agents. Improved knowledge about the risks is needed to identify opportunities to prevent work-related infectious diseases. The objective of the current study was to provide an updated overview of the published evidence on the exposure to pathogens among non-healthcare workers. Because of the recent SARS-CoV-2 outbreaks, we also aimed to gain more evidence about exposure to several respiratory tract pathogens.

**Methods:**

Eligible studies were identified in MEDLINE, Embase and Cochrane between 2009 and 8 December 2020. The protocol was registered with International Prospective Register of Systematic Reviews (CRD42019107265). An additional quality assessment was applied according to the Equator network guidelines.

**Results:**

The systematic literature search yielded 4620 papers of which 270 met the selection and quality criteria. Infectious disease risks were described in 37 occupational groups; 18 of them were not mentioned before. Armed forces (n=36 pathogens), livestock farm labourers (n=31), livestock/dairy producers (n=26), abattoir workers (n=22); animal carers and forestry workers (both n=16) seemed to have the highest risk. In total, 111 pathogen exposures were found. Many of these occupational groups (81.1%) were exposed to respiratory tract pathogens.

**Conclusion:**

Many of these respiratory tract pathogens were readily transmitted where employees congregate (workplace risk factors), while worker risk factors seemed to be of increasing importance. By analysing existing knowledge of these risk factors, identifying new risks and susceptible risk groups, this review aimed to raise awareness of the issue and provide reliable information to establish more effective preventive measures.

**Key messages**

**What is already known about this subject?**

- Employees in different types of work may be in several ways exposed to biological agents: more or less accidentally, through animal contact or by contact with other humans.

**What are the new findings?**

- Many non-healthcare workers also have evidence of exposure to infectious pathogens; several new occupational groups and pathogens are described. Exposure to respiratory tract pathogens was mentioned in 30 out of37 (81.1%) non-healthcare occupations that met our inclusion criteria. Many of these respiratory tract pathogens are readily transmitted where employees congregate (*workplace* risk factors).

**How might this impact on policy or clinical practice in the foreseeable future?**

- A combined risk factors approach (*disease* and workplace and *worker* risk factors) may result in a comprehensive risk assessment strategy. More research is needed on the impact of workplace (eg, crowding, exposure to dust and welding fumes) and worker (eg, age and immunosuppression) risk factors to obtain a more systematic approach to prevent biological risks among non-healthcare employees.

## Introduction

Work-related diseases accounted for 2.4 million (86.3%) of the total estimated deaths attributed to work in the updated report for the International Labour Organisation (ILO), published in 2017.^1^ Fatal occupational injuries accounted for the remaining 13.7%. The estimated fatal work-related mortality by cause in the year 2015, mentioned in the same report, was as follows: circulatory diseases (31%), work-related cancers (26%), respiratory diseases (17%) and occupational injuries (14%). Communicable diseases counted for 9% and were more common in low-income countries. They constituted slightly more than 30% of the work-related mortality in the African region vs less than 5% in high-income countries. The attributable fraction for infectious diseases was highest for women, both in high-income countries and other WHO regions (high-income region: men, 4.8%, vs women, 32.5%, and for the other regions: men, 3.1%, vs women, 20.7%). Morbidity from work-related infectious diseases is expected to be much higher, although the true extent of incident cases is difficult to establish due to under-reporting.^2^ Educational interventions to increase this reporting of occupational diseases by physicians have been studied by a former systematic review.^3^

According to the WHO, work-related diseases have multiple causes, where factors in the work environment may play a role, together with other risk factors, in the development of such diseases. On the other hand, an occupational disease is any disease contracted primarily as a result of an exposure to risk factors arising from work activity. Occupational exposure is defined as exposure to potentially harmful chemical, physical or biological agents that occurs as a result of occupational factors. Only a small subset of biological agents—pathogens—may cause disease in humans. Infectious diseases can be transmitted via direct contact (including percutaneous), droplet, airborne (bioaerosol), vehicles (such as food, water and fomites) and vectors. Transmission of biological agents in the workplace may occur in two directions: workers can acquire infections in the workplace and then also may serve as vectors that spread the disease to others, such as clients and coworkers. Occupations involving interaction with subgroups of the general population, particularly infected persons, pose an increased risk of infection. Disease transmission patterns are also relevant to those whose work brings them in contact with animals, putting them at risk of zoonotic infections.^2 4^

Since the former key review of Haagsma *et al*,^2^ not only *new occupations* are noticed but also *new pathogens* like SARS-CoV-2. Also, some occupations (eg, welding) might increase susceptibility of workers to infection on exposure to an infectious agent, without increasing the exposure to the pathogen per se.^5 6^

In the current pandemic of the infectious disease COVID-19, the Belgian Centre for Occupational Disease Risks (Fedris) registered 7930 declarations for healthcare workers and 79 declarations for employees in other, essential sectors (police inspectors, warehouse worker–food salesmen and firefighters) up to 13 October 2020.^7^ Indeed, not only healthcare workers are affected by the pandemic SARS-CoV-2 virus. Although the majority of the earliest patient cases reported possible zoonotic or environmental exposure at the Huanan Seafood Wholesale Market in Wuhan, it is now clear that human-to human transmission has been occurring.^8^ Koh described a case report among staff in the tourism, retail and hospitality industry, transport and security workers, and construction workers in Singapore.^9^ Recently, the EFFAT (European Federation of Trade Unions, in the Food, Agriculture and Tourism) reported outbreaks in slaughterhouses and meat processing plants in several European countries (Germany, Ireland, The Netherlands, the UK, France, Poland, Italy, Norway, Spain, Belgium and Denmark).^10^ According to preliminary research findings, the following risk factors have been identified: lack of physical distancing and inspections, poor housing conditions, shared transport, insufficient ventilation, lack of (adequate) personal protective equipment and colder temperatures.

Although there is an increasing number of publications regarding emerging infections such as SARS-CoV-2, few are related to occupational health, especially among non-healthcare workers and over a wider geographical area. Moreover, such studies could contribute to evidence of new risk factors (eg, infectious bioaerosols) for acquiring infections in exposed groups. This will be crucial in the development of effective interventions to prevent transmission of potentially zoonotic or other pathogens.^11^

### Objectives of this study

The objective of the current study was to provide an updated overview of the published evidence on the exposure to infectious pathogens in occupational groups other than healthcare workers. The second aim was to list significant work-related risk factors, including studies describing increased susceptibility to certain biological agents. By reason of the recent SARS-CoV-2 outbreaks, a third aim was to gain more evidence about exposure to respiratory tract pathogens among non-healthcare occupational groups.

## Methods

### Introduction

This systematic review was performed according to the guidelines of the Preferred Reporting Items for Systematic Reviews and Meta-Analyses (http://www.prisma-statement.org). The objective was formulated using the PICOS criteria (PICOS: *population*: non-healthcare workers; *intervention/exposure*: exposures to environmental processes which involve many different microorganisms (composting, recycling and waste water recycling), through animal contact (agriculture and food processing) or through contact with humans; *comparison*: non-exposed workers or general population; *outcome (primary)*: prevalence, incidence and/or occurrence rate of symptomatic infectious disease and/or seroconversion and/or immune-related and respiratory conditions; *outcome (secondary)*: independent risk factors; and *study*: observational studies including cohort studies, case–control studies, cross-sectional studies, outbreak reports and case series (three or more cases).To avoid unnecessary duplication, the protocol was sent to the International Prospective Register of Systematic Reviews database and registered under the number CRD42019107265 (http://www.crd.york.ac.uk/PROSPERO/). Duplicate records were checked by EndNote V.X7.

### Definitions

This study focused on biological agents such as bacteria, viruses, parasites or fungi, and was limited to work-related infectious disease, that is, infectious disease that is caused through work-related exposure or exacerbated by work-related factors.^12^ The excluded healthcare occupations were the following: dental care workers, healthcare assistants, nurses and midwife (assistant), hospital dietary workers, laboratory workers, medical doctors (and students) and microbiologists. Childcare workers were also excluded because they have a care-related job too. Funeral service workers were excluded because they may have the same infectious disease risks as mortuary workers in hospitals, while veterinary doctors and assistants were excluded because some countries (eg, The Netherlands), have separate vets for companion animals (dogs and cats) and farm animals (cows and horses). Only in the latter case, they have the same risks as farmers. *Biological agents* that are non-infectious were excluded, such as moulds that can trigger allergies or produce toxins. Thus, lung diseases caused by sensitisation or toxic reactions through inhalation of non-infectious bioaerosols (eg, hypersensitivity pneumonitis and organic dust toxic syndrome) were excluded. Mite infestation by *scabies* was included in the case of an outbreak, as well as colonisation by antimicrobial resistant pathogens. Infections that were contracted outside working hours (eg, HIV and other sexually transmitted infections among truck drivers or armed forces) were excluded. The study focused both on workers in *industrialised countries* as well as on workers in *low-income countries*, and also included infectious disease risks through *work-related* travel, for instance, among armed forces posted overseas, or airline personnel. These workers might be exposed to increased risk of infection compared with the population of their country of origin, because of endemic infections in the country of destination. Of specific consideration is that many factors may *combine* to increase the risk of infection among workers during pathogen transmission. Categories of these risk factors for work-related infections include *disease* factors (such as transmission mode), *workplace* factors (workplace characteristics, work practices and processes, and engineering and administrative issues), and *worker* factors (impaired immunity, inadequate prophylaxis, and socioeconomic and language factors).^4^

### Literature search

First, SC performed a *scoping review* of published papers in PubMed between January 2009 and December 2017 based on the search strategy employed by Haagsma *et al*,^2^ which was extensively documented in the published report and its appendices. Second, for the updated *systematic review* of Haagsma *et al* until 8 December 2020, an extensive electronic search strategy in Medline, Ovid, Embase.com and Cochrane CENTRAL was developed in collaboration with JAH and librarian WMB who have broad experience with systematic reviews. Because this systematic search strategy yielded more than 30 000 publications, the search terms were restricted by only screening the titles and major Medical Subject Headings (MeSH) terms, to include only articles where occupational diseases and infections were part of the major MeSH terms, or where these terms were mentioned in the title. The entire search profile is shown in the online supplemental appendix review 1.

10.1136/oemed-2020-107164.supp1 Supplementary data

### Inclusion criteria

Publications included in the review had to meet the following inclusion criteria:

1. The study (or at least an abstract) was published in the period of 1 January 2009–8 December 2020.
2. Work-related (occupational) exposure.
3. The study concerned employees of a specific occupational group (age≥16 years); gender, language, ethnicity were not considered as inclusion/exclusion criteria.
4. The study concerned specified infectious pathogen(s).
5. Symptomatic infectious disease and/or seroconversion and/or immune-related and/or respiratory conditions were used as outcome.
6. The exposure-associated risk for disease and/or seroconversion and/or immune-related and/or respiratory conditions was estimated by comparison to an appropriate reference population (for outbreak reports and case series, no reference group was needed).

### Data extraction

Relevant papers were screened independently in two rounds by SA (systematic review, full period) and SC (scoping review, until December 2017). SA performed a double check of all titles screened by SC. Differences were resolved by discussion with experts (eg, ADS). JAH screened also the first 10% of the systematic review, which led to the exclusion of review papers. Six additional publications were added by experts. In the first round of the systematic review, the title and abstract were taken into consideration and compared with the inclusion criteria, based on the review by Haagsma *et al*. In case the titles and abstracts did not provide enough information, the articles were moved forward to the second round. In the second round, the title, abstract and the full text were assessed.

### Quality assessment

An additional quality assessment was applied by SA in the third round according to the applicable reporting guidelines: Scottish Intercollegiate Guidelines Network (SIGN)^13^ for case–control and cohort studies, Strengthening the Reporting of Observational Studies in Epidemiology^14^ for cross-sectional studies, Outbreak Reports and Intervention Studies of Nosocomial Infection^15^ for outbreak reports and Case Report Guidelines^16^ for case series.

### Synthesis of study results

Results were first arranged in tabular form, listing all reported associations between pathogens and job titles or broader occupational groups. The International Standard Classification of Occupation (ISCO) of the ILO V.08 was used to classify job titles. ISCO classifies these job titles in four levels of aggregation in order to provide internationally comparable occupational data in a globalised market. We used level 3, which distinguishes 130 broader occupational groups. For some specific job titles, or where very specific associations were reported, level 4 subclassifications were used (table 1). Both levels are further defined as *occupational groups*. Subsequently, all occupational groups and their related pathogens were classified with their significant risk factors (online supplemental appendix review 2), and a random sample (systematic with random start, by SA in XLSTAT 2020) of pathogens with their corresponding disease, workplace and worker risk factors in table 2.

10.1136/oemed-2020-107164.supp2 Supplementary data

**Table 1:** Pathogens by specific job title or broader occupational groups

Occupational group	ISCO code	Pathogen
Abattoir workers and related food preparers	7511	(Methicillin-resistant) *Staphylococcus aureus*, (swine **(H3N2/H1N2)** influenza virus, **(avian) influenza virus (H9/H9N2), Avian metapneumovirus**, ***Bacillus anthracis***, ***Brucella abortus*** spp, *Campylobacter* spp, ***Chlamydia psittaci***, *Coxiella burnetii*, *Escherichia coli*, ***Francisella tulariensis*** **(=)**, hepatitis B virus, hepatitis E virus, ***Leptospira borgpetersenii/*** *hardjo/**interrogans/**pomona*, **Rift Valley fever virus**, **SARS-CoV-2 virus**, ***S. aureus* (=)**, *Streptococcus pyogenes*, *Toxocara canis*, ***Toxoplasma gondii*** **(=)**
Airline personnel	5111	Hepatitis E virus, **measles morbilivirus**
Animal carers	5164	*Bartonella hensalae*, *Borrelia burgdorferi*, ***B. canis***, *Capillaria hepatica*, *C. psittaci*, ***C. burnetii***, hantavirus (=), (**canine H3N8 (=**)) influenza virus, *Leptospira* spp, **lymphocytic choriomeningitis virus**, **mouse retroviruses (XMRV (=)/MLV (=**)), simian foamy virus, simian parvovirus, simian type D retrovirus, *T. canis*, *T. gondii* (≈)
Archaeologists	211	*Coccidioides immitis*
Armed forces	0000	(**Methicillin-susceptible) *S. aureus*, adenovirus (7/11A/B), astrovirus, chikungunya virus, *C. pneumoniae***, **coxsackie virus (A6), *C. burnetii***, **dengue virus, ECHO virus, hepatitis A/B/C** (≈)**/E virus, influenza A(H1N1/H3N2/H1N1pdm09)/B virus, *Legionella* spp,** *Leishmania* spp, ***Leptospira*** **spp**, **measles morbilivirus, *Microsporum canis, Mycobacterium tuberculosis*, mumps rubulavirus, norovirus, *Orientia tsutsugamushi*, *Plasmodium falciparum/ovale/vivax,* respiratory syncytial virus, Ross River virus, *non-typhoidal Salmonella enteretica,* sapovirus, *Sarcoptes scabiei*, SARS-CoV-2 virus, *S. pneumoniae*, *S. pyogenes*, *Trypanosomi cruzii* (=), *Yersinia enterocolica***
**Bar workers**	513	**HIV (=**)
**Barbers**	5141	**Hepatitis B virus (=**)
**Building workers**	711	***C. immitis*, *Histoplasma capsulatum***
**Cash collectors**	523	***M. tuberculosis***
**Civil engineering labourers**	9312	***Legionella pneumophila***
Cleaners	515	Hepatitis A virus, hepatitis B virus, *M. tuberculosis*
Divers	7541	*Campylobacter jejuni*, enteroviruses, *Pseudomonas aeruginosa*
Farm workers, crops	6111	*B. burgdorferi*, *Clostridium tetani,* ***C. immitis*,** *C. burnetii*, *E. coli, **F. tularensis**, Leishmania* spp, ***L. borgpetersenii/*** **spp,** *Strongyloides stercoralis,* **tick-borne encephalitis virus (=)**, **Toscana virus (=),** *T. canis,* **usutu virus (=), West Nile virus (=**)
**Firefighters**	5411	***Cryptosporidium parvum***
Fishmongers	7511	*Anasakis simplex*, **hepatitis E virus**
Forestry workers	6210	*Anaplasma phagocytophilum, **B. henselae***, *B. burgdorferi/**miyamotoi***, *C. burnetii*, ***Francisella tularensis***, hantavirus, **hepatitis E virus**, ***Leptospira* spp (=),** *Rickettsia conorii*, *R. helvetica*, tick-borne encephalitis virus ( ≈ ),**Toscana virus (=),** *T. gondii*, **usutu virus (=), West Nile virus (=**)
Gardeners	6113	*F. tularensis*
**Hotel workers**	9112	***L. pneumophila***
Livestock and dairy producers	6121	(Methicillin-resistant) *S. aureus*, **extended pectrum β-lactamase** (**≈/AmpC-producing** *E. coli*, (**equine (H3N8 (=))**/swine/avian (**H4 (=)/H5 (=)/H6 (=)/H7 (=)/H8 (=)/H9 (=)/H10 (=)/H11 (=)/H5N1/H5N2/H11N1/H9N2/H7N9**) influenza virus, ***B. anthracis***, *B. burgdorferi* ( ≈ ), *Brucella* spp (≈), *Campylobacter* spp, *C. psittaci*, *C. tetani*, *C. burnetii*, ***Crimean-Congo haemorrhagic fever virus**, Helicobacter pylori,* hepatitis E virus, ***Leishmania* spp**, *L. icterohaemorrhagiae*/**spp**, *M. bovis,* **Rift Valley fever virus** , ***Salmonella*** **spp** ( ≈ ), **severe fever with thrombocytopenia syndrome virus,** *S. suis*, *S. stercoralis, T. canis*, *T. gondii* ( ≈ ), West Nile virus
Livestock farm labourers	9212	(Methicillin-resistant) *S. aureus*, (**multidrug-resistant) *S. aureus,* (methicillin-resistant) *coagulase-negative staphylococci*, *extended spectrum **β**-lactamase/AmpC-producing*** *E. coli, **STEC O157/non-(STEC) O157,*** (avian (**H4 (=)/H5 (=)/H6 (=)/H7 (=)/H8 (=)/H9 (=)/H10 (=)/H9N2/H5N2/H7N3/H11N1/H5N1/** **H7N9**)/swine(**H2N3 (=)/H3N2v/H1N1/H3N2/(H1N1)pdm09)/H1N2**) influenza virus, ***Aspergillus flavus, A. fumigatus***, *B. burgdorferi*, *Brucella* spp, *Campylobacter* spp, ***Candida albicans***, *C. psittaci*, *Clostridium* spp*, *Clostridium tetani*, *C. burnetii*, ***C. parvum***, *H. pylori*, hepatitis E virus, *L. icterohaemorrhagiae*, *Moraxella* spp*, *M. bovis*, *Prevotella* spp*, ***R. conorii, R. felis***, **Rift Valley fever virus** , ***non-typhoidal S. enteretica***, *S. stercoralis*, *T. canis*, *T. gondii*, West Nile virus
**Manicurists**	5142	**Hepatitis B virus (=), hepatitis C virus (=), HIV (=**)
**Mining and mineral processing plant operators**	811	**(Panton-Valentine leucocidin-producing methicillin-susceptible)** ***S. aureus, Leptospira*** **spp, Marburg virus, measles morbillivirus,** ***M. tuberculosis***, ***Sporothrix schenckii***
**Office clerks**	4110	**Mumps rubulavirus**
Plant and machine operators and assemblers (metal and textile/leather)	812, 815	***B. anthracis, C. burnetii***, *H. capsulatum, L. pneumophila,* **measles morbillivirus, mumps rubulavirus,** *M. chelonae, **N. meningitidis C***, ***S. enteritidis***, ***S. pyogenes,*** **norovirus**
**Police officers**	5412	**HIV (=), mumps rubulavirus, varicella zoster virus**
Prison guards	5413	*M. tuberculosis*
**Professional drivers (bus or taxi**)	8322	***Legionella* spp, *M. tuberculosis***
Sex workers (female, **male**, **cis or transgender, internet escort**)	5168	*C. trachomatis*, hepatitis B virus ( ≈ ), hepatitis C virus (≈), herpes simplex virus-**2**, HIV ( ≈ ), human papilloma virus (**type 6/16/18/31/33/35/39/45/51/52/53, 56/58/59/66/67/68**), human T-lymphotrophic virus, *Neisseria gonorrhoeae*, *Treponema pallidum* (≈), *Trichomonas vaginalis*
**Ship’s stewards**	5111	**SARS-CoV-2 virus**
**Shop salespersons**	522	**SARS-CoV-2 virus**
**Social workers**	3412	***M. tuberculosis, Shigella sonnei***
Teachers, primary	2341	Cytomegalovirus, *N. meningitidis*
**Technicians**	313	**Mumps rubulavirus**
**Television crew**	265	***C. immitis***
Waste collectors	9611	***Blastocystis hominis* (=),** *Brucella* spp, ***C. burnetii***, ***Cryptosporidium*** **spp (=),** ***Entameuba histolytica*** **(=),** ***Giardia intestinalis*** **(=),** *H. pylori*, hepatitis A virus, hepatitis B virus, hepatitis C virus (≈), **hepatitis E virus (=), *Leptospira* spp (=)**, *T. gondii*
Wastewater workers	3132	***(Antibiotic-resistant) coagulase-negative staphylococci*, *(methicillin-resistant) S. aureus* (=), *methicillin-susceptible S. aureus (=), vancomycin-resistant enterococci (=), vancomycin-susceptible enterococci*, *H. pylori (=)*, hepatitis A virus** ( ≈ ), **hepatitis E virus** ( ≈ ), ***H. capsulatum***
**Welders**	7212	***S. pneumoniae***

Marked in bold are occupational groups or pathogens that were not yet described by studies in the former review of Haagsma *et al*.^2^ Pathogens with a possible portal of entry by inhalation (via the respiratory tract) are highlighted.

=, no increased risk when compared with a control group from the general population; ≈, some studies revealed no increased risk while other studies showed an increased risk.

*Nasopharyngeal microbiota content.

ISCO, International Standard Classification of Occupation; MRV, murine leukemia viruses; STEC, Shiga-toxin producing *Escherichia. coli* ; XMRV, xenotropic murine leukemia virus-related virus.

**Table 2:** Significant risk factors by described pathogens (the full list of references per pathogen is available in the online supplemental appendix review 2)

Pathogen	Disease factor	Workplace factor	Worker factor
Avian influenza virus	Moderate poultry exposure (301–900 poultry-years)^21^ Professional classification as a poultry seller^22^ ≥10 years of occupational exposure^23^	Another stall nearby, number of cages (more than five)^24^ Workplaces near locations where H5N2 outbreaks in poultry were reported^25^ Wholesale/retail live poultry markets^26^	Female gender^22 23^ Male gender^27 28^
*Coxiella burnetii*	Contact with small ruminants (sheep and goats)^29^ Cattle contact at own or other farm^30^ Keeping sheep or goats, exposure to arthropod bites^31^ ≥3 daily goat-related tasks (milking, feeding, supply and removal, general animal healthcare and birth assistance), other goat breeds next to white dairy goat^32^ Milking cattle, general healthcare of cattle, birth assistance, contact with raw milk, contact with cattle manure, contact with dead-born animals^33^	Presence of cat(s) in goat stable, distance residence to nearest stable ≤10 m, distance to nearest positive farm 0–<4 km^28^ Passed through the stores^34^	Full working week, worked in cattle sector in the past^30^ Age >50 years), rural area of residence, having little or no formal education^31^ Lived as child on a ruminant farm, no farm boots for staff^32^ Male gender^34^ No respiratory protection mask^35^ Living in rural areas^36^
Hepatitis E virus	Abattoir work, sewage work^37^ Occupational contact with animals (forestry/pig farm workers)^38^ Slaughterers^39^ Exposure to soil, contact with swine^40^ Having professions with exposure to pigs for more than 16.5 years^41^	Unorganised swine farming^37^ Woodcutting^42^ Raw seafood processing^43^ Feeding of pigs^44^ Previous mission abroad (military forces)^45^	Consumption of pork-liver sausages^37^ Residence area^40^ Age≥50 years, age group 25–34 years, ascending age, ages 40–49, 50–59, ≥60 and over 40 years^38 44 46–48^ ≥7 working years^43^ Living in an area with frequent flooding, consuming intern pig organs more than twice per week^49^ Ever been in Africa^50^
*Streptococcus pneumoniae*		Occupational exposure to welding fumes, silica dust^5^	

## Results

After removal of duplicates, 4620 unique results that met our search terms and time period were obtained. In the first round (scanning the titles and abstracts), 1369 articles were retained, while 3251 articles were excluded because they did not meet our inclusion criteria. In the second round, 932 articles were excluded after reading the full text, based on the same criteria. In the third, qualitative synthesis round, another 167 articles were excluded, resulting in 270 eligible studies. Observational studies including cohort, cross-sectional studies, case–control, outbreak reports and case series (three or more cases) were the included study designs.

**Figure 1:**
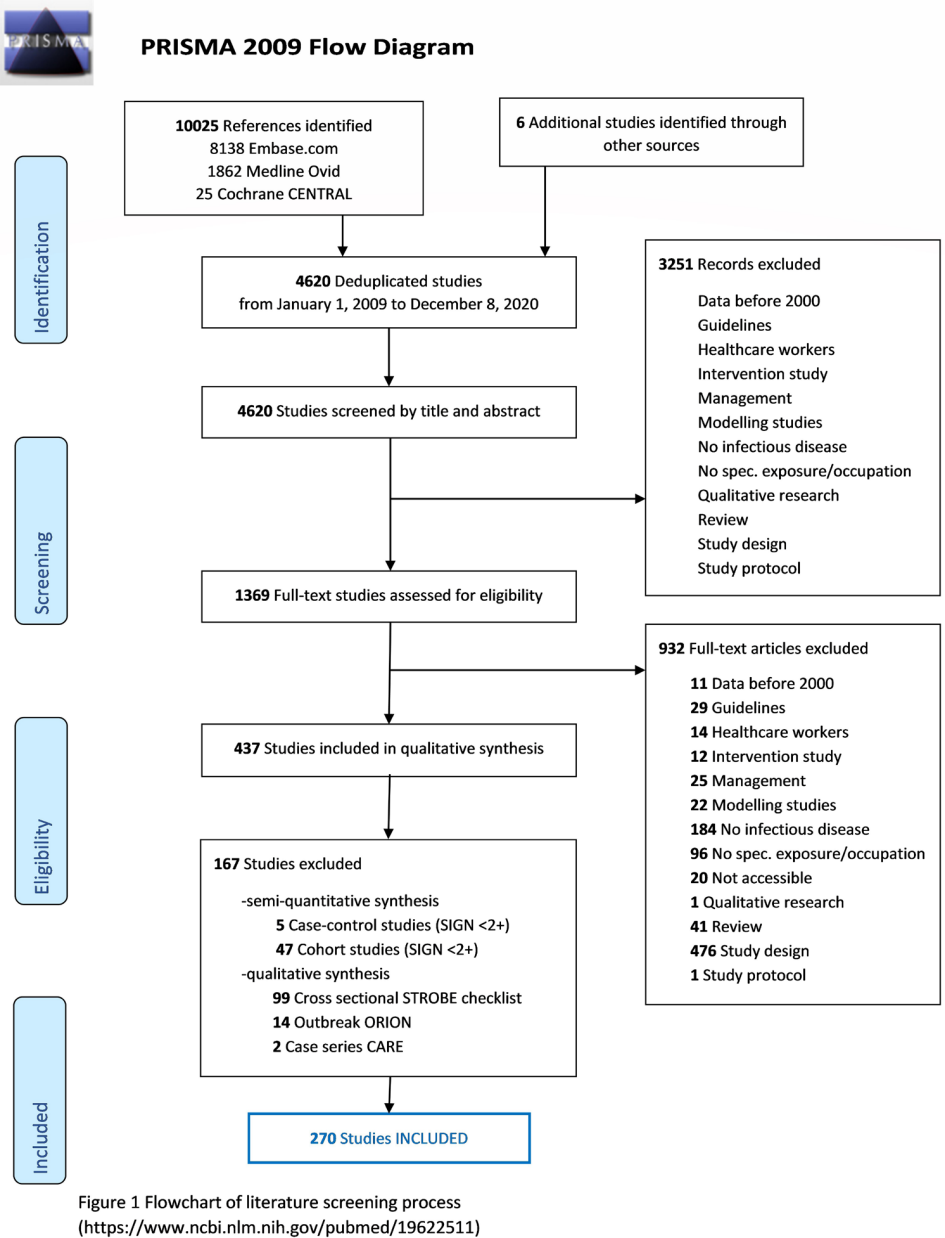
Flowchart of the literature screening process (https://www.ncbi.nlm.nih.gov/pubmed/19622511). CARE, Case Report Guidelines; ORION, Outbreak Reports and Intervention Studies of Nosocomial Infection; PRISMA, Preferred Reporting Items for Systematic Reviews and Meta-Analyses; SIGN, Scottish Intercollegiate Guidelines Network; STROBE, Strengthening the Reporting of Observational Studies in Epidemiology.

Figure 1 shows a flowchart of the literature screening process.

The remaining 270 full-text articles were systematically reviewed on job title, occupational group (ISCO-08 codes) and associated pathogens. The results are summarised in table 1. The literature review identified 37 occupational groups (classified by 38 ISCO-08 codes, at least at level 3) that were at risk of infectious disease. Studies describing infectious disease risks among 18 ‘new’ occupational groups (not listed by the earlier review of Haagsma *et al*) met our inclusion criteria (marked in bold in table 1). The occupational groups which were most frequently reported on exposure to different pathogens were armed forces (n=36 pathogens), livestock farm labourers (n=31 pathogens), livestock/dairy producers (n=26 pathogens), abattoir workers (n=22 pathogens), animal carers and forestry workers (both n=16 pathogens). Altogether, occupational exposures to 111 different pathogens (on genus or, if available, species level) were found, some of which were overlapping between occupational groups. One out of three pathogens (n=43, on genus or species level, marked in bold in table 1) were not yet described by studies in the earlier review of Haagsma *et al* (eg, avian metapneumovirus, chikungunya virus, Crimean-Congo haemorrhagic fever virus, dengue virus, equine influenza virus, Marburg virus, *Orientia tsutsugamushi*, SARS-CoV-2 virus and *Streptococcus pneumoniae*). Our recent update from 18 April to 8 December 2020 yielded another 752 publications, whereof 426 were COVID-19 related. After the third, qualitative synthesis round, five COVID-19 publications (four outbreak reports and one cross-sectional study) and eight non-COVID-19 publications met our inclusion criteria.

The mapping of studies per world region was as follows: Europe And Central Asia (n=75), East Asia and Pacific (n=67), North America (n=38), sub-Saharan Africa (n=33), Latin America and Caribbean (n=28), Middle East and North Africa (n=18) and South Asia (n=11). Thus, most studies were administered in Europe and Central Asia (27.8%), followed by East Asia and Pacific (24.8%), North America (14.1%) and sub-Saharan Africa (12.2%) (https://ourworldindata.org/world-region-map-definitions).

Online supplemental appendix review 2 includes a list of all reported associations between pathogens, job titles or broader occupational groups, and statistically significant risk factors separately for each article included in the review.

Exposure to respiratory tract pathogens (through human, animal or environmental pathways) was mentioned in 30 out of 37 (81.1%) of included occupational groups: abattoir workers and related food preparers, airline personnel, animal carers, archaeologists, armed forces, building workers, cash collectors, civil engineering labourers, cleaners, farm workers (crops), forestry workers, gardeners, hotel workers, livestock and dairy producers, livestock farm labourers, mining and mineral processing plant operators, office clerks, plant and machine operators and assemblers, police officers, prison guards, professional drivers (bus or taxi), ship’s stewards, shop salespersons, social workers, teachers, technicians, television crew, waste collectors, wastewater workers and welders.

Table 2 summarises combined, significant risk factors for pathogens with a global occurrence (avian influenza virus and *Coxiella burnetii);* work-related and travel-related infection risk (hepatitis E virus); or a single known risk factor (*S. pneumoniae*). Risk factors were subdivided in disease, workplace and worker risk factors. The full list of significant risk factors per pathogen is available in the online supplemental appendix review 2. For example, for *S. pneumoniae* infection, a single workplace risk factor (exposure to welding fumes and silica dust) was described by a recent study of Torén *et al*,^5^ while risk of hepatitis E virus infection through work-related travel, was mentioned for missions abroad among military forces (workplace factor). For hepatitis E virus, avian influenza virus and *C. burnetii*, additional disease and worker risk factors were described in several studies.

## Discussion

Work-related infectious diseases among non-healthcare workers include a wide variety of pathogens and occupational groups. Almost half of the listed occupational groups and one out of three listed pathogens were not yet described by studies in the earlier review of Haagsma *et al*.^2^ This is in accordance with the 2015 European Working Conditions Survey, in which an increasing proportion of European workers (13%, which is 1.5 times as many as 10 years earlier) were indicated to be exposed to infectious agents at work.^17^ Although most of the reported studies in the current review were of European and Central Asian origin (27.8%), some of the infectious disease risks were limited to certain geographical regions (eg, *Coccidioides immitis* in North and Latin America and Rift Valley fever virus in sub-Saharan Africa). Other infectious disease exposures are reported globally (eg, *Mycobacterium tuberculosis*, *Histoplasma capsulatum* and *S. pneumoniae*). However, worker susceptibility may vary per region. These *worker* risk factors (age, gender, inadequate prophylaxis, and socioeconomic and language factors) seem to be of increasing importance, as seen in the increased number of papers reporting these worker risk factors over the last 10 years. Also, many immunosuppressant drugs (biologicals, glucocorticoids, antimetabolite drugs and inhibitors of cytokine production and function) and diseases (eg, HIV, stem cell or organ transplantation) result in impaired immunity and thus increase susceptibility to infectious disease risks, without increasing the exposure to the pathogen per se. More research in this domain is needed because it is estimated that a high number of employees work under this condition.^18^ Furthermore, migrant workers may also have an increased risk of infectious diseases in high-income countries due to language barriers, different prophylactic vaccination strategies and employment by several contractors. This results in a difficult implementation of outbreak control measures as illustrated in the recent *S. pneumoniae* outbreak on a shipyard in France.^19^ A combined risk factors approach may result in an extended risk assessment strategy based on the former exposure matrix of Haagsma *et al*,^2^ by combining human, animal and environmental transmission pathways (*disease risk factors*) with their corresponding *workplace* risk factors (eg, contact with dust, welding fumes and crowded work environment) and *worker* risk factors (eg, poor nutritional status, immunosuppression, pregnancy, smoking, viral coinfections and comorbidity).^4 19 20^ Exposure to respiratory tract pathogens was mentioned in 30 out of 37 (81.1%) of included non-healthcare occupational groups, indicating that biological hazards such as *M. tuberculosis* (among armed forces, cash collectors, cleaners, miners, prison guards, professional drivers and social workers), measles (armed forces, operators and miners) and SARS-CoV-2 virus (armed forces, meat processing workers, retail workers and ship’s stewards) are not limited to healthcare workers and thus must be included in the risk analysis. Many of these respiratory tract infections are readily transmitted where employees congregate, for example, in transportation vehicles, correctional facilities, military barracks, slaughterhouses and meat-packing plants, and shipyards (*workplace* risk factors). Of special interest is the global occurrence of antimicrobial-resistant pathogens among abattoir workers, livestock dairy producers and farm workers, while the risk among wastewater workers seemed to be low. Most of these exposures lead to colonisation, which can lead to an infection in the case of a health event or weakened immune system conditions. One major limitation of this study is that the literature review was restricted to papers published in the period 2009–8 December 2020, while a strength might be that the findings since the year 1999 by the former review by Haagsma *et al* were also included. However, established and important occupationally induced infections that were recognised prior to 1999 may not have been included in more recent publications and consequently in this review because they are no longer new findings. On the other hand, studies are still being conducted on recent hazards such as the SARS-CoV-2 virus. Furthermore, the search profile may not have yielded all articles on occupational exposure to infectious disease, due to the focus on non-healthcare workers and the terms used in the search strategy. Including the search term ‘respiratory conditions’ as outcome, yielded much more records and subsequently infectious diseases risks among military forces. In light of this limitation, we recommend further exhaustive searches using the exposure matrix proposed in Haagsma *et al* and other sources such as the Latin American and Caribbean Health Sciences Literature Database (Lilacs http://lilacs.bvsalud.org/). Another limitation is that we did not include mortality or hospitalisation studies because they did not meet our inclusion criteria. In the first place, we wanted to provide an overview of risk factors for disease. Risk factors for mortality (and also for hospitalisation) could possibly be checked in a subsequent review, also because there are probably other/supplementary risk factors for these outcomes. Because there was a large heterogeneity in occupational groups (n=37), pathogens (n=111), measures of effect and study designs (eg, outbreak reports, n=84) in the studies that met our inclusion criteria, it was not appropriate to conduct a meta-analysis. Another limitation of our study was that the occupational aspects in many published papers (in non-occupational journals) are often downplayed by the authors, resulting in information and selection bias. In contrast to the earlier review by Haagsma *et al*,^2^ also studies reporting non-significant differences or lower risks in the same occupational risk were included, in order to try to minimise potential publication (selection) bias. In addition, the condition ‘symptomatic infectious disease and/or seroconversion’ was extended with ‘immune-related and/or respiratory conditions’ to enhance inclusion of immune-related diseases and respiratory tract pathogens. Still, due to reporting and selection biases, the included occupational groups are almost certainly not representative of the whole set of non-healthcare occupations. By adding the qualitative screening step, more information could be gained about the strength of causality, the precision of the estimated association between exposure and outcome, and the independence of risk factors. For example, cohort studies were, according to the SIGN criteria,^13^ only included if they were prospective; only studies that reported values with confidence limits were included. Some trends which were observed during the screening process might be of interest. First, 716 studies (51.1% of the assessed full texts) were excluded because of inadequate study design, specifically lack of comparison of incidence or prevalence to an adequate reference population. Certainly, some of the studies in this group were never designed to answer the question of occupational risk. For example, many studies of HIV in sex workers addressed the effectiveness of interventions or differential prevalence and incidence in subgroups of sex workers (eg, outdoor sex work, cisgender vs transgender, internet escorts). These studies were clearly not designed to answer the question of occupational risk in the entire group of sex workers. Nevertheless, they were highly abundant among the studies excluded based on criterion 5 (eg, 71.3% of the excluded intervention studies were on HIV). On the other hand, an important proportion of studies excluded based on criterion 5 were designed specifically to answer the question of occupational risk but did not include a comparison to an adequate reference population. Second, evidence from the former Cochrane review by Curti *et al* 3 indicated that only a small number of occupational infectious diseases were reported to the designated registration systems, while Haagsma *et al* already stated that blood-borne pathogens were more frequently reported compared with zoonoses.^2^ As a result, the present body of literature might give an incomplete and to some extent unbalanced overview of occupational risks due to infectious diseases. In addition, it is difficult to compare national data due to differences in compensation criteria. The recent SARS-CoV-2 virus outbreaks draw attention to these types of occupational risk. For example, not only the healthcare workers but also uniformed service occupations (eg, police officers and firefighters) and other crucial sectors and essential services (cleaners, food industry and abattoir workers) were considered *at-risk* professions by Fedris during the lockdown period.^7^ Third, some occupational groups which involve travel abroad (eg, military personnel) might expose these workers to increased risk of infection (eg, hepatitis E virus) compared with the population of their country of origin, because of endemic infections in the country of destination. A decision needs to be made on the criteria which need to be fulfilled in order for such situations to be classified as an occupational infectious disease risk. For example, a consideration could be whether these workers have higher infectious disease risk than the local population (eg, because of lack of immunity or unadjusted behaviour). Fourth, a distinction could be made between occupation risk for endemic pathogens versus emerging (epidemic) infections. In the latter case, the occupational infection risk could be assessed through modelling approaches or by reference to historical outbreaks of emerging pathogens with similar biological and epidemiological characteristics.

## Conclusion

Two main groups of biological agents of relevance for occupational health could be recognised. The first group comprised infectious diseases, including, but not limited to, zoonotic infections, for which certain occupational groups are at increased risk. The second group comprised organisms which, when present in the work environment, result in the production of bioaerosols. These bioaerosols can be either non-infectious (eg, endotoxins) or infectious (eg, influenza and measles virus). Within this domain, this review was restricted to infectious bioaerosols. Exposure to respiratory tract pathogens was mentioned in 81.1% of non-healthcare occupational groups that met our inclusion criteria. Many of these respiratory tract pathogens are readily transmitted where employees congregate, for example, in transportation vehicles, correctional facilities, military barracks, slaughterhouses and meat-packing plants, and shipyards (*workplace* risk factors). Currently, more research is needed on the impact of these workplace risk factors (eg, crowding, exposure to dust and welding fumes) and also on *worker* risk factors (eg, age and immunosuppression) to obtain a more systematic approach to preventing biological risks among non-healthcare employees. This combined risk factors approach (*disease*, *workplace* and *worker* risk factors) may result in an extended risk assessment strategy. By analysing existing knowledge of these risk factors, identifying new risks and susceptible risk groups, this review aimed to raise awareness of the issue and provide reliable information that can support efforts to establish effective preventive measures.
