# Supplementary material for: Global infectious disease risks associated with occupational exposure among non-healthcare workers: a systematic review of the literature
Source: Occup Environ Med. 2021 May 25;79(1):63–71. doi: 10.1136/oemed-2020-107164 (PMC8685622; doi:10.1136/oemed-2020-107164)
Supplement: Supplementary data [file oemed-2020-107164supp001.pdf]

Table A1. Total number of references of published articles from January 1, 2009 to December 8, 2020

infectious diseases occupational exposure

	References	References after deduplication
embase.com	8251	8138
Medline Ovid	7060	1862
Cochrane CENTRAL	94	25
**Total**	**15405**	**10025**

embase.com

(infection/mj/exp OR 'environmental microbiology'/mj/de OR 'virus'/mj/exp OR 'virus transmission'/mj/exp OR 'bacterium'/mj/exp OR 'parasite'/mj/exp OR 'helminth'/mj/exp OR 'fungus'/mj/exp OR 'microorganism'/mj/exp OR 'infectious agent'/mj/exp OR 'infection risk'/mj/exp OR 'infection control'/mj/exp OR 'bloodborne bacterium'/mj/exp OR 'coronavirus disease 2019'/de OR 'Severe acute respiratory syndrome coronavirus 2'/exp OR (((communicable) NEAR/3 disease*) OR infect* OR zoonos* OR zoonot* OR parasit* OR mycos* OR virus OR viral OR bacter* OR ((environment* OR air) NEAR/3 microbiol*) OR parasite* OR helminth* OR fungus OR fungi OR fungal OR microorganism* OR micro-organism* OR pathogen OR pathogens OR mould OR bioaerosol* OR bio-aerosol* OR seroconversion* OR hiv OR mrsa OR malaria OR cmv OR tubercul* OR q-fever OR staphylococ* OR enterococ* OR Leptospir* OR Cytomegalovir* OR seropositive* OR Brucell* OR hepatit* OR tetanus OR lyme OR Borrelia* OR influenza OR dengue OR legion* OR hcv OR hpv OR papilloma* OR meningit* OR aids OR covid OR coronavirus-disease-2019 OR sars-cov-2 OR Severe-acute-respiratory-syndrome-coronavirus-2):ti) AND ('work'/mj/de OR 'work environment'/mj/de OR 'workroom air'/mj/de OR workplace/mj/de OR 'employee'/mj/exp OR personnel/mj/de OR worker/mj/de OR 'laboratory personnel'/mj/de OR 'nonmedical occupations'/mj/exp OR 'occupational disease'/mj/exp OR 'occupational health'/mj/exp OR 'occupational medicine'/mj/exp OR 'occupational exposure'/mj/exp OR 'industrial hygiene'/mj/exp OR (at-work OR ((worker OR workers) NOT (care-worker* OR hospital-worker* OR medical-worker* OR health-worker* OR healthcare-worker*)) OR laborer* OR labourer* OR occupation* OR (employee* NOT (care-employee* OR hospital-employee* OR medical-employee* OR health-employee* OR healthcare-employee*)) OR workplace OR (personnel* NOT (care-personnel* OR health-personnel* OR hospital-personnel* OR medical-personnel* OR healthcare-personnel*)) OR butcher* OR hunter* OR veterinarian* OR farmer* OR slaughterhouse OR slaughter-house OR abbatoir* OR barber* OR (Waste NEAR/3 Picker*) OR veteran OR veterans OR (police NEAR/3 officer*) OR soldier* OR teacher* OR (truck NEAR/3 driver*) OR ((working OR work) NEAR/3 (environment* OR associate* OR farm* OR industr*)) OR fishermen* OR military OR ((food OR meat) NEAR/3 handl*) OR (cattle NEAR/3 keeper*) OR miners OR farmworker*):ti) AND ('risk factor'/mj/exp OR 'risk'/mj/exp OR 'risk assessment'/mj/exp OR 'statistics'/mj/exp OR 'epidemiological data'/mj/exp OR 'epidemiology'/mj/exp OR exposure/mj/de OR 'occupational exposure'/mj/de OR 'environmental exposure'/mj/de OR 'statistics and numerical data'/mj/de OR (risk OR risks OR statistic*

OR numerical* OR epidem* OR seroepidem* OR incidence* OR prevalen* OR seroprevalen* OR expos* OR burden* OR emerging OR mobidit* OR mortalit* OR surviv* OR outbreak*):ti) NOT ([Conference Abstract]/lim) AND [English]/lim

Medline Ovid

(*Infection/ OR *Environmental Microbiology/ OR exp *Viruses/ OR exp *Virus Diseases/ OR exp *Bacteria/ OR exp *Parasites/ OR exp *Helminths/ OR exp *Fungi/ OR *Infection Control/ OR COVID-19/ OR SARS-CoV-2/ OR COVID-19.nm. OR severe-acute-respiratory-syndrome-coronavirus-2.nm. OR (((communicable) ADJ3 disease*) OR infect* OR zoonos* OR zoonot* OR parasit* OR mycos* OR virus OR viral OR bacter* OR ((environment* OR air) ADJ3 microbiol*) OR parasite* OR helminth* OR fungus OR fungi OR fungal OR microorganism* OR micro-organism* OR pathogen OR pathogens OR mould OR bioaerosol* OR bio-aerosol* OR seroconversion* OR hiv OR mrsa OR malaria OR cmv OR tubercul* OR q-fever OR staphylococ* OR enterococ* OR Leptospir* OR Cytomegalovir* OR seropositive* OR Brucell* OR hepatit* OR tetanus OR lyme OR Borrelia* OR influenza OR dengue OR legion* OR hcv OR hpv OR papilloma* OR meningit* OR aids OR covid OR coronavirus-disease-2019 OR sars-cov-2 OR Severe-acute-respiratory-syndrome-coronavirus-2).ti.) AND (*Occupational Groups/ OR *Work/ OR *Workplace/ OR *Laboratory Personnel/ OR *Occupational Health Services/ OR *Occupational Diseases/ OR *Occupational Health/ OR *Occupational Medicine/ OR *Occupational Exposure/ OR (at-work OR ((worker OR workers) NOT (care-worker* OR hospital-worker* OR medical-worker* OR health-worker* OR healthcare-worker*)) OR laborer* OR labourer* OR occupation* OR (employee* NOT (care-employee* OR hospital-employee* OR medical-employee* OR health-employee* OR healthcare-employee*)) OR workplace OR (personnel* NOT (care-personnel* OR health-personnel* OR hospital-personnel* OR medical-personnel* OR healthcare-personnel*)) OR butcher* OR hunter* OR veterinarian* OR farmer* OR slaughterhouse OR slaughter-house OR abbatoir* OR barber* OR (Waste ADJ3 Picker*) OR veteran OR veterans OR (police ADJ3 officer*) OR soldier* OR teacher* OR (truck ADJ3 driver*) OR ((working OR work) ADJ3 (environment* OR associate* OR farm* OR industr*)) OR fishermen* OR military OR ((food OR meat) ADJ3 handl*) OR (cattle ADJ3 keeper*) OR miners OR farmworker*).ti.) AND (*Risk Factors/ OR *Risk/ OR *Risk Assessment/ OR *Epidemiology/ OR *Occupational Exposure/ OR *Environmental Exposure/ OR (risk OR risks OR statistic* OR numerical* OR epidem* OR seroepidem* OR incidence* OR prevalen* OR seroprevalen* OR expos* OR burden* OR emerging OR mobidit* OR mortalit* OR surviv* OR outbreak*).ti.) AND english.la.

Cochrane CENTRAL

((((communicable) NEAR/3 disease*) OR infect* OR zoonos* OR zoonot* OR parasit* OR mycos* OR virus OR viral OR bacter* OR ((environment* OR air) NEAR/3 microbiol*) OR parasite* OR helminth* OR fungus OR fungi OR fungal OR microorganism* OR micro next organism* OR pathogen OR pathogens OR mould OR bioaerosol* OR bio next aerosol* OR seroconversion* OR hiv OR mrsa OR malaria OR cmv OR tubercul* OR q next fever OR staphylococ* OR enterococ* OR Leptospir* OR Cytomegalovir* OR seropositive* OR Brucell* OR hepatit* OR tetanus OR lyme OR Borrelia* OR influenza OR dengue OR legion* OR hcv OR hpv OR papilloma* OR meningit* OR aids OR covid OR coronavirus NEXT disease NEXT 2019 OR sars NEXT cov NEXT 2 OR Severe NEXT acute NEXT respiratory NEXT syndrome NEXT

coronavirus NEXT 2):ti) AND ((at next work OR ((worker OR workers) NOT (care next worker* OR hospital next worker* OR medical next worker* OR health next worker* OR healthcare next worker*)) OR laborer* OR labourer* OR occupation* OR (employee* NOT (care next employee* OR hospital next employee* OR medical next employee* OR health next employee* OR healthcare next employee*)) OR workplace OR (personnel* NOT (care next personnel* OR health next personnel* OR hospital next personnel* OR medical next personnel* OR healthcare next personnel*)) OR butcher* OR hunter* OR veterinarian* OR farmer* OR slaughterhouse OR slaughter next house OR abbatoir* OR barber* OR (Waste NEAR/3 Picker*) OR veteran OR veterans OR (police NEAR/3 officer*) OR soldier* OR teacher* OR (truck NEAR/3 driver*) OR ((working OR work) NEAR/3 (environment* OR associate* OR farm* OR industr*)) OR fishermen* OR military OR ((food OR meat) NEAR/3 handl*) OR (cattle NEAR/3 keeper*) OR miners OR farmworker*):ti) AND ((risk OR risks OR statistic* OR numerical* OR epidem* OR seroepidem* OR incidence* OR prevalen* OR seroprevalen* OR expos* OR burden* OR emerging OR mobidit* OR mortalit* OR surviv* OR outbreak*):ti)
