# Supplementary material for: Global infectious disease risks associated with occupational exposure among non-healthcare workers: a systematic review of the literature
Source: Occup Environ Med. 2021 May 25;79(1):63–71. doi: 10.1136/oemed-2020-107164 (PMC8685622; doi:10.1136/oemed-2020-107164)
Supplement: Supplementary data [file oemed-2020-107164supp002.pdf]

Table A2. Summary of described job titles or occupational groups, pathogens and statistically significant risk factors separately for each article included in the review

**Author**	**Job title or Occupational group**	**Pathogen**	**Significant risk factors**
2010[1]	Military forces (Navy)	Influenza A virus (H1N1)*	NA
Abseno[2]	City bus drivers and cash collectors	Mycobacterium tuberculosis	None identified.
Adjemian[3]	Miners	Marburg virus*	NA
Alavi [4]	Rice farmers	Leptospira spp.	None identified.
Allard[5]	Building workers	Histoplasma capsulatum*	NA
Alonso[6]	Waste-sorting workers	Coxiella burnetii*	No respiratory protection mask (OR=2.8, 95%CI 1.2-6.6)
Alonso[7]	Pet transport couriers	Coxiella burnetii*	NA
Al-Sayyad[8]	Police and Military recruits	Mumps virus*	NA
Al-Thaqafy[9]	Military forces (National Guard)	Hepatitis B virus	Older age (aOR=5.35, 95%CI 1.15-25.01 for those aged ≥35 years compared with those aged <25 years); presence of a household member with HBV (aOR=2.35, 95%CI 1.06-5.19); and previous endoscopy (aOR=2.55, 95%CI 1.06-6.11)
Alvarado[10]	Military forces (Navy)	SARS-CoV-2 virus*	NA
Alvarado-Esquivel[11]	Waste pickers	Leptospira spp.(=)	None identified.
Alvarado-Esquivel[12]	Meat workers (abattoir, butcher)	Leptospira spp.	Rural residence (aOR= 4.29, 95%CI 1.29-14.20), duration in the activity (aOR=3.87, 95%CI 1.13 - 13.22), snake meat consumption (aOR=7.20, 95%CI 1.48-34.91), consumption of unwashed raw fruits (aOR=6.68, 95%CI 1.16-38.55)
Alvarado-Esquivel[13]	Meat workers	Toxoplasma gondii(=)	None identified.
Alvarado-Esquivel[14]	Livestock raiser, animal hair dresser	Toxoplasma gondii(=)	Eating while working (OR =7.14, 95%CI 1.91-26.72), consumption of duck meat (OR=5.43, 95%CI 1.43-20.54)
Ambrose[15]	Military forces	Legionella spp.*	Occupants of the building

			closest to the cooling tower (aOR=6.9, 95%CI 2.2-22)
Amsalu[16]	Medical waste handlers	Hepatitis B virus, hepatitis C virus (=)	Age older than 40 years (OR=2.77, 95%CI 1.08-7.12), working in a hospital laundry (OR= 9.41, 95%CI 1.06-83.84)
Andriopoulos[17]	Livestock farmers	Brucella spp.(=)	None identified.
Ansari-Moghaddam[18]	Municipal waste workers	Hepatitis B virus	Exposure to waste (aOR= 9.36, 95%CI 2.01-43.7); lack of vaccination against HBV (aOR=3.83, 95%CI 1.86-25.2); jaundice (aOR=6.91, 95% CI 1.51-31.5); history of endoscopy (aOR=2.86, 95%CI 1.08-7.62); and high risk behaviors (aOR=4.80, 95%CI 1.96 - 27.2)
Aquino[19]	Military forces (Navy)	Influenza A virus (H3N2)*	NA
Archer[20]	Farmers	Rift Valley Fever virus*	NA
Armstrong[21]	Tunnel workers	Histoplasma capsulatum*	NA
Awah-Ndukum[22]	Abattoir workers	Brucella spp.	Handling of foetus and uterine contents (OR=13.00, 95%CI 1.51-111.88)
Awosanya[23]	Kennel workers	Leptospira spp.	Contact with sewage (OR=15.0, 95%CI 1.5-759); presence of wounds or cuts on either hands or legs (OR=12.0, 95%CI 1.7-147.5)
Bailey[24]	Armed forces	Leishmania spp.*	Proximity of accommodation to the cleared scrub (OR= 6.6, 95%CI 2.3-19.1 for front row versus other two rows)
Bansal[25]	Abattoir workers, sewage workers, swine workers	Hepatitis E virus	Sewage work (OR=31.25, 95%CI 7.78-125.52), abattoir work (OR=25.00, 95%CI 6.81-64.74), unorganized swine farming (OR=26.85, 95%CI

			8.68-83.10)
Banta[26]	Military trainees	Coxsackievirus A6*	NA
Baral[27]	Female sex workers	HIV	None identified.
Barnhart[28]	Female bar workers	HIV(=)	NA
Beaudoin[29]	Swine workers	Swine influenza A virus (H2N3)(=)	NA
Beheshti[30]	Abattoir workers	Brucella spp.	None identified.
Bellali[31]	Farmers	Leishmania spp.	Missed the irrigation tour (aOR=2.34, 95%CI 1.10-4.95); avoid administrative procedures (aOR=2.74, 95% CI 1.30-5.79)
Bernier[32]	Female sex workers	HIV	Outdoor sex work (aOR=3.29, 95%CI 1.72-6.27); primary residence (other Russian region than Moscow) (aOR=2.61, 95%CI 1.05-6.48)
Beste[33]	Military Forces	Hepatitis B virus	Service in a combat zone (aOR=1.56, 95%CI 1.01-2.41); being wounded in combat (aOR=1.79, 95%CI 1.04-3.08)
Bilman[34]	Factory workers	Salmonella enteritidis*	NA
Birku[35]	Military forces	Hepatitis B virus, Hepatitis C virus (=)	Age ≥ 40 years (OR=7.6, 95%CI 2.0-29.0); history of nose piercing (OR=5.9, 95%CI 1.2-29.9); sexually transmitted infection (OR=4.3, 95%CI 1.1-16.4)
Boost[36]	Abattoir workers (pigs)	MRSA	None identified.
Borkenhagen[37]	Swine workers	Swine influenza virus (H1N1 and H3N2)	Swine H1N1: working at swine confined animal feeding operations (aOR= 19.16, 95%CI 3.55-358.65). Swine H3N2: working at swine confined animal feeding operations (aOR=2.97, 95 CI 1.16-8.01), outbreak among animals in the last 30 days (aOR=8.62, 95%CI 1.87-61.79)
Boscarino[38]	Military Forces	Hepatitis C virus	Other exposures (e.g.

			vaccinations or shots in the military in the 1960s) (OR=2.63, 95%CI 2.00-3.46)
Boyce[39]	Female sex workers	HIV	Entered the sex trade under age 16 years (aOR= 4.6, 95%CI 1.6-13.2); had not received HIV education in their first year of sex trade (aOR=2.8, 95%CI 1.5-5.5), had experienced violence to force commercial sex (aOR=4.6, 95%CI 2.2-9.8); had not used condoms in their first month (aOR= 2.8, 95%CI 1.3-6.1)
Brinker[40]	Military forces	Leptospira spp.*	NA
Brooks[41]	Animal handlers (mice)	Mouse retroviruses (XMRV (=), MLV (=))	NA
Brosh-Nissimov[42]	Military forces	Microsporum canis*	Female gender (aOR= 3.92, 95%CI 1.68-9.17), animal contact at Base (aOR=2.9, 95%CI 1.09-7.73), performed guarding duty (aOR=6.37, 95%CI 2.2-18.44)
Cárcamo[43]	Female sex workers	Neisseria gonorrhoeae, HIV, Syphilis, Trichomonas vaginalis, Chlamydia trachomatis, HSV2	None identified.
Carpentier[44]	Forestry workers	Hepatitis E virus	Age ≥50 years (aOR=4.54, 95%CI 2.64-7.84); woodcutting (aOR=2.24, 95%CI 1.33-3.77)
Caruso[45]	Swine farmers	Hepatitis E virus	None identified.
Cassir[46]	Shipyard workers	Streptococcus pneumoniae*	NA
Cavaretto[47]	Manicurists	Hepatitis B virus (=), hepatitis C virus (=), HIV (=)	NA
Chabata[48]	Female sex workers	HIV	None identified.
Chapman[49]	Military forces	Norovirus*	Drinking/brushing teeth with water from source other than water trailer (OR=2.7, 95%CI 1.66-4.27); cleaning bathrooms (OR=4.6, 95%CI 1.69-12.55)
Chaudhry[50]	Butchers	Avian influenza (H9)	Another stall

			nearby (aOR=3.38, 95%CI 1.78-6.39); number of cages (more than 5) (aOR=4.9, 95%CI 1.60-14.97)
Chaussade[51]	Forestry workers, pig farm workers	Hepatitis E virus	Consumption of pork-liver sausages (aOR=4.48, 95%CI 2.63-7.64). Forestry workers: occupational contact with animals (OR=1.58, 95%CI 1.03-2.43). Pig farm workers: occupational contact with animals (OR=2.51, 95%CI 1.70-3.70)
Chen[52]	Factory workers	Streptococcus pyogenes*	Age (18–20 year) (p<0.05), roommate or teammate with Streptococcus pyogenes colonization (p<0,05)
Cheng[53]	Military trainees	Adenovirus type 7*	NA
Choi[54]	Live animal market employees	Swine influenza (H3N2v)	None identified.
Coman[55]	Agriculture workers	Avian influenza virus (H9N2)	Moderate poultry exposure (301-900 poultry-years) (aOR=3.6, 95%CI, 1.1-12.1)
Cook[56]	Abattoir workers	Leptospira spp.	Having wounds (aOR=3.1, 95%CI 1.5-6.1), smoking (aOR=1.8, 95%CI 1.1-2.9); eating at work (aOR=2.1, 95%CI 1.2-3.6), cleaning the offal (aOR= 5.1, 95%CI 1.8 to 15.0), and having a borehole for personal water use (aOR=2.3, 95%CI 1.1-4.7)
Cook[57]	Abattoir workers	Rift Valley Fever virus	Being the slaughterman (aOR=3.5, 95%CI 1.0-12.1)
Cosby[58]	Military forces	Influenza virus (H3N2)*	NA
Cowan[59]	Police recruits	Varicella zoster virus*	NA
Crowell[60]	Men who sell sex to men	HIV	Age > 30 years (aRisk Ratio= 1.40, 95%CI 1.14-1.72), female gender identity (aRisk Ratio = 1.40, 95%CI 1.23-1.59), unknown education level

			(aRisk Ratio = 1.95, 95%CI 1.31-2.91)
Crucitti[61]	Female sex workers	Trichomonas vaginalis	None identified.
Cui[62]	Seafood processing workers	Hepatitis E virus	Raw seafood processing (aOR=3.82, 95%CI 2.54-5.8), ≥7 working years (aOR=3.69, 95%CI 2.16-6.31)
Cummings[63]	Construction workers	Coccidioides immitis*	NA
Das[64]	Construction workers	Coccidioides immitis*	NA
De Keukeleire[65]	Farmers	Borrelia burgdorferi	None identified.
De Keukeleire[66]	Forestry workers	Borrelia burgdorferi	Use of protection measures (aOR=1.57, 95%CI 1.15-2.15), intensity of tick bites (number, frequency) (aOR=3.66, 95%CI 2.40-5.57)
De Lange[67]	Sheep farmers	Coxiella burnetii	Cattle contact at own or other farm (aOR=3.87, 95%CI 2.13-7.04), full working week (aOR=2.42, 95%CI 1.13-5.15), worked in cattle sector in the past (aOR=1.79, 95%CI 1.01-3.18)
De Laval[68]	Military Forces	Norovirus*	Consumption of raw mussels (RR=11.2, 95% CI 1.7-74.8)
De Marco[69]	Swine workers	Swine influenza virus (H1N1)	None identified.
De Souza[70]	Female sex workers	Treponema pallidum	Low level of education attending up to the primary school (aPR=3.8, 95%CI 1.4-9.2); high frequency of anal sex during the past year (aPR =9.3, 95%CI 3.5-28.7)
Delgado Naranjo[71]	Machine tool workers	Coxiella burnetii*	NA
Di Renzi[72]	Forestry workers	Borrelia burgdorferi	None identified.
Diabate[73]	Female sex workers	Neisseria gonorrhoeae, Chlamydia trachomatis, HIV	HIV-positive FSWs (treated: aHR=4.2, 95%CI 1.60-10.77); untreated: aHR=4.2, 95%CI 1.59-11.49) were more likely to acquire NG/CT compared to HIV-negative FSWs. Place of work (bars,

			hotels, other) (aHR=2.4, 95%CI 1.19-4.68) compared to brothels. No consistent condom use (aHR=2.0, 95%CI 1.11-3.72)
Dongliu[74]	Military forces	Adenovirus type B*	NA
Dreyfus[75]	Abattoir workers (sheep)	Leptospira hardjobovis, Leptospira borgpetersenii	Working in the offal room (aOR=6.5, 95% CI 1.4-29.8), working at the middle and end of the slaughter floor (aOR=8.2, 95%CI 2.1-32.7), working at the beginning of the slaughter floor (aOR=10.4, 95%CI 2.8-38.8)
Dreyfus[76]	Abattoir workers (sheep)	Leptospira interrogans, Leptospira borgpetersenii	None identified.
Duggan[77]	Pet fancy rats workers	Hantavirus (=)	NA
Eassa[78]	Municipality Solid-Waste Workers	Cryptosporidium (=), Entameuba histolytica (=), Giardia intestinalis (=), Blastocystis hominis (=)	Direct exposure to solid fecal waste (OR= 1.8, 95%CI 1.1-3.0), occupational activities that allowed for direct exposure to solid fecal waste (OR=2.3, 95%CI 1.4-4.0), rural residence (aOR=1.9, 95%CI 1.1-3.2)
Esmaeili[79]	Butchers and slaughterhouses workers	Brucella spp., Coxiella burnetii	Brucella.: having a work history of more than 15 years risk (OR=2.00, 95% CI 1.02-3.90), contact with small ruminants (sheep and goats) (OR=2.36, 95%CI 1.06-5.25). Coxiella: contact with small ruminants (sheep and goats) (OR=7.07, 95%CI 2.04-24.50)
Ferreira-Junior[80]	Female sex workers	HIV, Treponema pallidum, hepatitis B and C	None identified.
Fragaszy[81]	Swine workers	Influenza A virus (H1N1)pdm09	None identified.
Geenen[82]	Poultry workers	MRSA	None identified.
Geng[83]	Abattoir workers (rabbit)	Hepatitis E virus	None identified.
Gobet[84]	Military forces	Mumps virus*	NA
Goldstein[85]	Water spray irrigators	(Antibiotic-resistant)coagulase-negative staphylococci	None identified.

González[86]	Female sex workers	Human papillomavirus	Commercial sex work (RR= 4.72, 95%CI 2.45-9.09)
Govender[87]	Miners	Sporothrix schenckii *	≤3 years' mining (aOR=4.0, 95%CI 1.2-13.1)
Guerra[88]	Military forces	Plasmodium ovale	None identified.
Gutierrez Garitano[89]	Public institution workers	Shigella sonnei*	Canteen-food consumption (mOR=3.84, 95%CI 1.02-14.44)
Hargreaves[90]	Female sex workers	HIV	None identified.
Harris[91]	Military forces	Sarcoptes scabiei*	NA
Hatcher[92]	Industrial hog operation workers	Multidrug-resistant S. aureus (MDRSA)	None identified.
Hensen[93]	Female sex workers	HIV	No/incomplete primary education versus incomplete secondary education (aOR=1.79, 95%CI 1.16-2.75); started selling sex aged 10 to 14 versus started selling sex aged 20 to 24 (aOR=2.51, 95%CI 1.42-4.43)
Hoan[94]	Pig workers (abattoir, farm, vendors)	Hepatitis E virus	None identified.
Hinjoy[95]	Pig farmers	Hepatitis E virus	Living in an area with frequent flooding (aOR=1.64, 95%CI 1.00-2.68), consuming intern pig organs more than twice per week (aOR=3.23, 95%CI 1.15-9.01)
Ho[96]	Military forces	Norovirus*	NA
Huang[97]	Poultry workers	Avian influenza viruses (H5N2 and H7N3)	Poultry workers whose workplaces were near locations where H5N2 outbreaks in poultry were reported (aOR=5.6, 95%CI 1.5-20.8)
Huang[98]	Male sex workers	HIV (=), Treponema pallidum (=)	HIV: Syphilis infection (aOR=9.0, 95%CI 2.4-33.5)
Huijbers[99]	Poultry workers	Extended-spectrum/AmpC β-lactamase-producing Escherichia coli	Having diabetes or skin disease(s) (41.2% (yes) versus 15.8% (no); aOR=16.5; p=0.002), sampling in July–December 2010

			(33.3% versus 7.8% in January-May 2011; aOR=13.0; p=0.002)
Hulin[100]	Abattoir workers (poultry)	Chlamydia psittaci	None identified.
Hurtado[101]	Hoists and chains manufacturing	Coxiella burnetii*	NA
Hwang[102]	Military forces	Adenovirus*	NA
Iser[103]	Food processing plant workers	Neisseria meningitidis C*	Work at the processing plant (mOR=22, 95%CI 2.33-207.7), ≥ 20 people in work section (mOR=8, 95%CI 1.46-43.6). <u>Household factors</u>: no ventilation at home (mOR=14, 95%CI 1.07-176.4), >4 residents/house (mOR=8, 95%CI 1.01–62.8), ≥1 person in the same bed (mOR=12, 95%CI 1.24–115.4).
Isler[104]	Services for homeless	Mycobacterium tuberculosis	None identified.
Ismail[105]	Gold mine workers	Panton-Valentine leukocidinproducing methicillin-susceptible Staphylococcus aureus*	NA
Jahfari[106]	Forestry workers	Borrelia miyamotoi	None identified.
Jeger[107]	Military forces	Influenza virus (H1N1)*	NA
Jia[108]	Female sex workers	Human papillomavirus	Post-menopause (aOR= 2.9, 95%CI 1.1-7.8)
Jurke[109]	Forestry workers	Bartonella henselae, Borrelia burgdorferi, Francisella tularensis, Leptospira spp.(=), Hantavirus, tick-borne encephalitis virus (TBE)	<u>Bartonella</u>: >10 h/week In the woods during leisure time (aOR=1.54, 95%CI 1.07-2.24). <u>Borrelia</u>: gender (male) (aOR=5.28, 95%CI 2.39-11.67), working professionally in the forest (mainly outdoors) (aOR=2.54, 95%CI 1.45-4.46), being ≥ 50 years (aOR=2.02, 95%CI 1.14-3.58), more than 50 tick bites (aOR= 2.04, 95%CI 1.39-2.99). <u>Hantavirus</u>: frequently

			deploy mouse trap (aOR= 2.40, 95%CI 1.17–4.94). Francisella: hunting hares/other, mixed and no hunting (aOR=8.93, 95%CI 2.09–38.11), working > 10 h/week near water (aOR=3.78, 95%CI 1.36-10.51), having touched a mouse (aOR= 3.86, 95%CI 1.52-9.81). TBE: go diving (aOR= 6.29, 95%CI 1.40-28.29)
Kajon[110]	Military forces	Adenovirus 11a*	NA
Kang[111]	Farmers (swine)	Hepatitis E virus	Residence area (aOR= 1.97, 95%CI 1.26-3.09), contact with swine (aOR= 3.46, 95%CI 2.32-5.17), exposure to soil (aOR= 3.58, 95%CI: 2.12-6.03)
Katelaris[112]	Raspberry workers	Leptospira borgpetersenii*	Interpreter required (aOR 4.00, 95%CI 1.63-9.86), saw rodents (aOR= 7.09, 95%CI 1.29-38.93)
Kato[113]	Assembly-line workers, office clerks, technicians	Mumps virus*	NA
Kayali[114]	Poultry workers (chicken)	Avian Influenza viruses (H4 and H11) (=)	None identified.
Kayali[115]	Poultry workers (turkey)	Avian influenza virus (H4/H5/H6/H7/H8/H9/H10) (=)	None identified.
Kayali[116]	Poultry workers (turkey)	Avian metapneumovirus	Meat-processing plant worker (aOR=4.1, 95%CI 1.3-13.1)
Kho[117]	Animal farm workers	Rickettsia conorii, Rickettsia felis	R. conorii: age over 50 years (43.6%, 95%CI 27.3%-59.9%) versus 41-50 years (7.7%, 95%CI 0.0%-16.4%, p=0.002). R. felis: age over 50 years (33.3%, 95%CI 17.9%-48.8%) versus ≤20 years old (5.4%±22.9%, p=0.015) and 31–40 years old (5.0%, 95%CI 0.0%-10.7%, p=0.009).
Khounvisith[118]	Pig farmers	Hepatitis E virus	Feeding of pigs (OR= 2.03, 95%CI 1.02- 4.05), age

			group 25–34 years (OR= 3.82, 95%CI 1.19–12.23)
Khurelbaatar[119]	Avian or horse workers	Avian (=) and equine influenza virus A (=)	NA
Kissling[120]	Wool-processing factory workers	Bacillus anthracis	Days working on machines processing raw goat hair without a mask (Prevalence ratio= 1.12, 95%CI 1.0-1.2, p=0.039)
Klumb[121]	Animal agriculture workers	Campylobacter spp., Cryptosporidium parvum, Shiga-toxin producing Escherichia coli (STEC) O157/non-(STEC) O157, non-typhoidal Salmonella enteretica (NTS)	Estimated cumulative incidence for people living and/or working on a farm per 10 000 population compared to other Minnesotans; for Campylobacter, 92.0 vs. 7.5 (p< 0.001); C. parvum, 23.8 vs. 1.2 (p< 0.001); NTS, 21.4 vs. 7.4 (p< 0.001); STEC O157, 5.4 vs. 1.1 (p< 0.001) and non-O157 STEC, 4.4 vs. 1.3 (p< 0.001).
Knust[122]	Animal care workers (mice)	Lymphocytic choriomeningitis virus*	NA
Kobayashi[123]	Airport workers	Measles morbilivirus*	NA
Krueger[124]	Animal care workers (dogs)	Canine influenza (H3N8)(=)	NA
Krueger[125]	Animal care workers (dogs)	Brucella canis	Kennel employees (aOR= 2.7, 95% CI 1.3-5.8)
Krumbholz[126]	Pig farmers and slaughterers	Swine influenza viruses	None identified.
Krumbholz[127]	Pig workers	Hepatitis E virus	Slaughterers (Prevalence= 41.7%, 95%CI 22.1–63.4, p=0.0090)
Kunwar[128]	Military forces	Dengue virus*	NA
Kupferman[129]	Abattoir workers	Leptospira interrogans*	NA
Lagler[130]	Military forces	Hepatitis E virus	Previous mission abroad (OR=2.49, 95%CI 1.74-3.58)
Lan[131]	Retail workers	SARS-CoV-2 virus	Direct customer exposure (aOR=5.1, 95% CI 1.1-24.8)
Laroucau[132]	Poultry workers(chicken)	Chlamydia psittaci*	NA
Larson[133]	Horse workers (veterinarians, breeders, others)	Equine influenza virus	Having worked as an equine veterinarian (aOR= 14.0, 95%CI 2.6-75.9),

			reporting a history of smoking (aOR=3.1, 95%CI 1.2-7.7), receipt of a seasonal influenza vaccine between 2000 and 2005 (aOR=2.3, 95%CI= 1.1-5.0)
Lee[134]	Swine farmers	Hepatitis E virus	Age (aOR=1.07 per year, 95%CI 1.05-1.09)
Lee[135]	Military forces	Salmonella non-typhi*	NA
Li[136]	Pig workers	Methicillin-resistant coagulase-negative staphylococci	Frequency of pig contact (days per week) (mean ratio=1.92, 95%CI 1.62-2.27), duration of pig contact (years) (mean ratio=1.50, 95%CI 1.34-1.69)
Li[137]	Poultry workers (feed, slaughter and sell poultry)	Avian influenza virus (H9N2)	Male (aOR=1.6, 95%CI 1.2-2.8)
Li[138]	Factory workers	Norovirus*	Drinking from direct water disposal (Risk Ratio=3.0, 95%CI 1.7-5.3), contact with infected colleagues (Risk Ratio=6.1, 95%CI 3.4-11.0)
Li[139]	Farmers	Severe fever with thrombocytopenia syndrome virus	Graving (aOR=17.98, 95%CI 4.35-74.26), grass mowing (aOR=14.05, 95%CI 5.53-35.70), raising cattle (aOR=11.51, 95%CI 2.18-60.67), time spent on farm work (aOR=3.24, 95%CI 1.49-7.05), tick bites (aOR=3.81, 95%CI 1.22-11.85), age (OR= 1.88, 95%CI 1.27-2.78)
Liang[140]	Swine farmers	Hepatitis E virus	Age 40-49 (OR=4.14, 95%CI 2.15-7.96), age 50-59 (OR=2.80, 95%CI 1.09-7.22), age ≥60 (OR= 6.75, 95%CI 2.60-17.54)
Linkevicius[141]	Shipyard workers	Streptococcus pneumoniae*	NA
Liu[142]	Military recruits	Influenza A and B virus*	NA
Liu[143]	Military forces	Ross river virus*	NA
Lopes-João[144]	Military forces	Norovirus, Astrovirus, Sapovirus*	NA

López-Robles[145]	Swine workers	Swine influenza virus (H3N2)	Farms with high number of breeding herd (aOR= 3.98, 95%CI 1.00-15.86)
Lord[146]	Abattoir workers	Coxiella burnetii*	NA
Ma[147]	Swine workers	Swine influenza virus (H3N2)	Developed a respiratory illness during the last 12 months (aOR=3.2, 95%CI 1.2-8.2), seasonal H3N2 (aOR=4.8, 95%CI 2.2-10.8)
Macdonald[148]	Military forces	Yersinia enterocolitica*	Consumption of mixed salad (aOR=10.26, 95%CI 0.85-123.57)
Marinho[149]	Recyclable waste collectors	Hepatitis B virus	Illicit drug use (Prevalence ratio=2.05, 95%CI 1.15-3.68), age over 40 years (Prevalence ratio=6.18, 95%CI 1.92-19.82)
Marinho[150]	Recyclable waste collectors	Hepatitis C virus (=)	A history of STIs (aOR= 6.7, 95% CI 1.2-36.5), unprotected sex with multiple partners (aOR= 7.6, 95%CI 0.9-66.0)
Marra[151]	Female sex workers	Human papilloma virus	Risk factors for vaginal hrHPV infection: being under 25 years of age (p= 0.010), being Dutch (p < 0.001), having an anal hrHPV infection (aOR= 10.50, 95%CI 7.73-14.26)
Martins[152]	Recyclable waste pickers	Hepatitis E virus (=)	Age over 40 years (aOR=5.2, 95%CI 1.5-17.5)
Matos[153]	Female sex workers	Hepatitis B virus, Hepatitis C virus (=)	Hepatitis B virus: age (aOR=1.13, 95%CI 1.08-1.19), previous blood transfusion (aOR=3.16, 95%CI 1.15-8.69), cocaine use (aOR=4.54, 95%CI 2.01-10.28), single (aOR= 3.80, 95%CI 1.69-8.54), ignoring burning pain during urination as a symptom of STI (aOR= 2.57, 95%CI 1.14-5.78), ignoring genital ulcers/sores as a symptom of STI (aOR= 3.47, 95%CI 1.58-7.58)

Mayet[154]	Military forces	Norovirus*	NA
Mayet[155]	Military forces	influenza A(H1N1)*	NA
Mayet[156]	Military forces	Measles Morbillivirus*	Age < 20 years (IRR=18.9, 95%CI 7.4-48.3), age 20-29 (IRR=8.3, 95%CI 3.9-18.0), age 30-39 years (IRR=2.8, 95%CI 1.2-6.4). Branch of armed forces: navy: IRR=1.8, 95%CI 1.0-3.1), army: IRR=1.9, 95%CI 1.2-3.0), logistical services: IRR=2.7, 95%CI 1.3-5.9)
Mbareche[157]	Swine workers	Nasopharyngeal flora microbiota	Multivariate analysis: air samples and nasopharyngeal flora of pig workers cluster together, compared to the non-exposed control group. The significance was confirmed with the PERMANOVA statistical test (p=0.0001)
McCurdy[158]	Farm workers	Coccidioides immitis	Self-reported dust exposure for most recent job (aOR=1.9, 95%CI 1.0-3.5), work with root and bulb vegetables in past year (aOR=3.0, 95%CI 1.2-7.1), work with carrots (aOR=2.9, 95%CI 1.0-8.6)
McKinnon[159]	Male sex workers	HIV	Frequency of sex with male partners (aHR=1.33/ sex act, 95%CI 1.01-1.75)
McKinnon[160]	Female sex workers	HIV	Minimum charge/sex act (aHR=5.7, 95%CI 1.96-16.59 for high versus intermediate), N. gonorrhoeae infection (aHR=5.89, 95%CI 2.03-17.08), sex with casual clients during menses (aHR=6.19, 95% CI 2.58-14.84), Depo Provera use (aHR=5.12, 95% CI 1.98-13.22), estimated number

			of annual unprotected regular partner contacts (aHR=1.004, 95%CI 1.001-1.006)
Méndez[161]	Military forces	Trypanosoma cruzi (=)	NA
Mirzazadeh[162]	Female sex workers	HIV, Treponema pallidum, Neisseria gonorrhoea, Chlamydia trachomatis, Trichomonas vaginalis, Human papilloma virus	HIV: Lifetime injection drug use (aOR=5.32, 95% CI 2.31-12.3); lifetime non-injection drug use (aOR=2.33, 95%CI 1.13-4.81); middle school and above education level versus illiterate (aOR= 0.22, 95%CI 0.08-0.61)
Moayedi-Nia[163]	Female sex workers	HIV, HCV, HBV, HSV, Treponema pallidum (=)	HIV infection: being infected with HSV type1/type2 (aOR=5.13, 95% CI 1.0-27.76), being under 25 years of age (aOR=4.51, 95%CI 1.0-21.49)
Mohd Ridzuan[164]	Plantation workers	Leptospira spp.	The presence of cows in plantations (aOR=4.78, 95%CI 2.76-8.26), the presence of a landfill in plantations (aOR=2.04, 95%CI 1.22-3.40)
Mol[165]	Domestic and healthcare waste handlers	Hepatitis B virus, Hepatitis C virus	Hepatitis B: age (aOR=1.06, 95%CI 1.02-1.11), current working time (years) (aOR=1.17, 95%CI 1.05-1.30) Hepatitis C: history of imprisonment (aOR=6.54, 95%CI 1.04-41.22)
Montuori[166]	Wastewater workers	Hepatitis A virus (=)	Shellfish consumption and duration of employment were significantly associated with positivity (p<0.01 and p< 0.05, respectively). The interaction term (age x duration of employment) was significant (p< 0.001), age (aOR=2.68; 95% CI 1.48-4.85)

Mor[167]	Male sex workers	STI/HIV	STI/HIV infection: low-risk men who have sex with men (a0R=0.1, 95%CI 0.02-0.7, versus the reference male sex workers)
Mossong[168]	Military forces	Mumps orthorubulavirus*	NA
Mostafavi[169]	Animal husbandry workers	Coxiella burnetii	Age > 50 years (OR=2.58, 95%CI 1.39-4.79), rural area of residence (OR=2.01, 95%CI 1.22-3.28), having little or no formal education (OR=7.78, 95%CI 3.29-18.39), keeping sheep or goats (OR=7.67, 95% CI 1.75-33.61), exposure to arthropod bites (OR = 2.34, 95% CI 1.39-3.96)
Mughini-Gras[170]	Swine workers	Hepatitis E virus	Ever been in Africa (aOR= 2.20, 95%CI 1.06-4.53)
Mulders[171]	Abattoir workers	MRSA	Working with live chickens: 'hanger'(aOR= 11.27, 95%CI 4.18-30.43), conventional electric stunning (reference=CO_2 stunning) (aOR=4.36, 95%CI 1.55-12.26)
Munseri[172]	Police officers	HIV (=)	NA
Muraguri[173]	Male sex workers	STI/HIV	Male sex workers were significantly more likely than non-sex workers to report victimization to verbal (57.7% versus 23.1%, p<0.001), physical violence (15.1% versus 0.8%, p<0.001), sexual violence (10.0% versus 2.5%, p=0.013)
Musyoki[174]	Female sex workers	HIV	Unrecognized HIV infection: Increasing age (30-34 years: aRR=2.4, 95%CI 1.3-4.3); 35-62 years (aRR=2.1, 95%CI 1.2-3.7), inconsistent condom use with paying clients (aRR=2.1, 95%CI

			1.4-3.0), use of a male condom as a method of contraception (aRR=2.5, 95%CI 1.7-3.5)
Mutagoma[175]	Female sex workers	Treponema pallidum, HIV, Hepatitis B virus (=), Hepatitis C virus (=)	Positive association with syphilis/HIV co-infection: age 25 years and older (aOR=1.82, 95% CI 1.33-2.50), having had a genital sore in the last 12 months (aOR=1.34, 95% CI 1.05-1.71), having HBsAg-positive test (aOR=2.09, 95%CI 1.08–4.08)
Narayanan[176]	Male sex workers	HIV, HSV-2	HIV: duration in sex work (aOR=1.08, 95%CI 1.03-1.13), Syphilis serology (aOR=3.83, 95%CI 1.36-10.79), HSV-2 status (aOR=12.92, 95%CI 7.00-23.84).<br>HSV-2: duration in sex work (aOR=1.19, 95%CI 1.11-1.27), HIV status (aOR=10.70, 95%CI 5.63-20.33)
National Institute for Infectious Diseases, Shinjuku-ku, Tokyo, Japan [177]	Ship's stewards	SARS-CoV-2*	NA
Navdarashvili[178]	Livestock workers	Bacillus anthracis	Slaughtering animals (aOR=7.3, 95% CI 2.9-18.1), disposing of dead animals (aOR=13.6, 95%CI 1.5-119.8)
Neo[179]	Military forces	Norovirus (group I.7 and II.17)*, Sapovirus (group II.3)*	NA
Ngoupa[180]	Barbers	Hepatitis B virus (=)	NA
Nguyen[181]	Poultry workers (chicken)	Extended-spectrum β-lactamase-producing -producing Escherichia coli(=)	Antimicrobial usage (during the month prior to the study visit) (aOR=2.52, 95%CI 1.08-5.87)
Nicas[182]	Construction workers	Coccidioides immitis*	NA
Nielsen[183]	Military forces	Mumps orthorubulavirus*	NA
Nielsen[184]	Livestock farming	Coxiella burnetii	Living in rural areas (RR=2.6; 95% CI 1.9-3.5)

Nivesvivat[185]	Military forces	Methicillin-susceptible Staphylococcus Aureus*	Risk factors of acquiring skin abscesses: multiple erythematous papules (aOR=3.4, 95%CI 1.5-7.5), training in subunit 1 (aOR=32.6, 95%CI 4.1-260.6), training in subunit 2 (aOR=26.9, 95%CI 3.3-218.2)
Okoye[186]	Poultry workers	Avian Influenza virus	Not identified.
Oliveira-Filho[187]	Female sex workers	Hepatitis C virus	Up to elementary school versus high school or more (aOR=2.3, 95%CI 1.3-6.3); up to one minimum wage versus more than one minimum wage (aOR= 5.5, 95%CI 1.7-16.8); illicit drug use (injectable or inhaled) (aOR=9.4, 95%CI 3.9-19.5); unprotected sex (aOR=32.1, 95%CI 10.8-74.3); more than five sexual partners (aOR=2.5, 95%CI 1.3-4.2); condom exemption for clients paying extra (aOR=14.2, 95%CI 4.9-28.4); more than seven years working in the sex trade (aOR=4.6, 95%CI 1.8-7.6); Changes in genitalia (wart, wound, and/or itching) (aOR=3.2, 95%CI 1.2-6.0); did not perform medical/gynecological examination (aOR=4.5, 95%CI 1.7-8.2)
Oré[188]	Military forces	Leishmaniasis spp.*	Being male (aRisk Ratio= 4.01, 95%CI 1.11-14.42), not wearing long-sleeve clothes (aRisk Ratio=1.71, 95%CI 1.18- 2.50), sleeping in open rooms (aRisk ratio=1.80, 95%CI 1.16-2.79)
Pang[189]	Military forces	Influenza A virus, Coxsackie/ ECHO virus	Febrile respiratory illness: increasing age (aOR= 1.03,

			95% CI 1.01-1.05), recruit camp (aOR=4.67, 95% CI 3.99-5.46), smoking (aOR= 1.31, 95% CI 1.13-1.52). Influenza A(H1N1)pdm09: Malay ethnicity (aOR=1.50, 95% CI 1.04-2.15). Coxsackie/ECHO virus: Malay ethnicity (aOR=1.67, 95% CI 1.19-2.36). Contact risk factors for FRI: stay-out personnel with ill household member (aOR=4.96, 95% CI 3.39-7.24), stay-in personnel with ill bunkmate and household member (aOR=3.55, 95% CI 2.57-4.91)
Park [190]	Female sex workers (cisgender)	Chlamydia trachomatis, Neisseria gonorrhoeae, Trichomonas vaginalis	Chlamydia: past year sex work initiation (aHR=2.7, 95%CI 1.3-6.0), Gonorrhea: past year sex work initiation (aHR=1.7, 95%CI 1.0-2.8), client sexual violence (aHR=2.9, 95%CI 1.2-7.1), Trichomonas: having female sexual partners (aHR=3.4, 95%CI 1.3-8.5)
Park[191]	Military forces	Respiratory Syncytial Virus*	NA
Parveen[192]	Miners	Leptospira spp.	Water bodies on the way (OR=10.6, 95%CI 2.86-39.54), wet surroundings of mine (OR=10.6, 95%CI 2.54-40.63), rat infestation (OR=4.6, 95% CI 1.17-18.01), cattle in mine (OR=10.4, 95%CI 2.15-50.27)
Patterson[193]	Female sex workers	HIV, Treponema pallidum, Neisseria gonorrhoeae, Chlamydia trachomatis	HIV or any STI: spouse has been diagnosed or treated for an STI past six months (aOR=2.97, 95%CI 1.32-6.68), most or all clients are from elsewhere than Mexico (aOR=1.91, 95%CI 1.13-3.22)

Pawar[194]	Poultry workers	Avian influenza (H9N2)	Male (aOR=1.6, 95%CI 1.2-2.8)
Pirnay[195]	Military forces	SARS-CoV-2 virus*	NA
Quan[196]	Poultry workers	Avian influenza (H9N2, H7N9)	H9N2 infection: female (aOR=1.6, 95%CI 1.2-2.1); poultry seller (aOR=1.9, 95%CI 1.4-2.6). H7N9 infection: female (aOR=2.2, 95%CI 1.4-3.6); poultry seller (aOR=4.1, 95%CI 2.2-7.7)
Rachiotis[197]	Municipal solid waste collectors	Hepatitis A virus	Age group > 42 years (aOR=22.57, 95%CI 7.29-69.88), education group ≤9 years (aOR=2.19, 95%CI 1.01-4.78), duration of employment ≥16 years (aOR=3.57, 95%CI 1.15–11.08)
Ramos[198]	Military forces	Streptococcus pyogenes*	Contact with another case (OR=6.92, 95%CI 3.60-13.30)
Raza[199]	Military forces	Sarcoptes scabiei	Itching in family/ colleagues (aOR=95.41, 95%CI 9.97-912.91), changing clothes < 2 times/week (aOR= 5.63, 95%CI 2.35-13.50), sharing beds (aOR=4.44, 95%CI 2.19-9.01), bathing < 1 time/day (aOR=4.32, 95%CI 2.25-8.29), low education (aOR=3.37, 95%CI 1.82-6.25), leave/temporary Duty (aOR=2.50, 95%CI 1.49-4.13)
Reeves[200]	Military forces	Chikungunya virus*, Dengue virus*	NA
Remoli[201]	Agricultural and forestry workers	Tick-borne encephalitis (=), Toscana virus (=), Usutu virus (=), West Nile virus (=)	NA
Ricci[202]	Civil engineering	Legionella pneumophila	Age group ≥50 years (aOR=3.11, 95%CI 1.57-6.16), aquatic sports (swimming, fishing, rowing, and others) (aOR=

			2.26, 95%CI 1.09-4.68)
Richter[203]	Poultry workers (turkeys)	MRSA	None identified.
Ringhausen[204]	Miners	Mycobacterium tuberculosis	Age ≥80 years (aOR=5.8, 95%CI 2.04-16.26), foreign country of birth (aOR= 6.8, 95%CI 1.91-24.0)
Rodkvamtook[205]	Military forces	Orientia tsutsugamushi*	NA
Rosenberg Goldstein[206]	Spray irrigation workers	MRSA (=), methicillin-susceptible S. aureus (=), VRE (=), vancomycin-susceptible enterococci	None identified.
Rossow[207]	Farming activities	Francisella tularensis	<u>Pneumonic tularaemia</u>: exposure to hay dust (aOR 6.6, 95%CI 1.9-25.4)
Royal[208]	Military forces	Coxiella burnetii	None identified.
Sahibzada[209]	Swine workers	CA-MRSA ST93	Persons working with farrowing sows (aOR= 6.39, 95%CI 1.23-39.36)
Sahneh[210]	Rice farmers	Leptospira spp.	Wet working environment (aOR=7.65, 95%CI 3.58-16.35), exposure to stagnant rice paddy water while having a skin scratch/injury (aOR=18 , 95%CI 5.00-63.00); washing the face with stagnant rice paddy water (aOR=11.83, 95%CI 3.74-37.43), and sighting of rats or rat nest in rice paddies (aOR=3.61, 95%CI 1.14-11.35)
Sakamoto[211]	Professional drivers	Legionella spp.	'Sometimes' use car air-conditioning (p=0.026, Fisher's exact probability)
Sanchez[212]	Military forces	Norovirus*	Consumption of cake prepared by Norovirus infected food worker (aOR=10.1, 95%CI 1.2-81.6), pizza (aOR= 3.6, 95%CI 1.1-11.9)
Sánchez-Anguiano[213]	Female sex workers	Chlamydia trachomatis	Consumption of alcohol (aOR=2.39, 95% CI 1.0-5.71)
Santos[214]	Sewer workers	Histoplasma capsulatum*	NA
Sargianou[215]	Farm labourers (sheep)	Crimean-Congo hemorrhagic	Former tick bite (aOR=

		fever virus	18.3, 95%CI 2.22-151), agro-pastoral occupation (aOR=6.99, 95%CI 1.01-48.4), living at an altitude of ≥ 400 m (aOR= 17, 95% CI 2.48-117)
Schimmer[216]	Dairy Goat Farmers	Coxiella burnetii	≥ 3 of daily goat-related tasks (milking, feeding, supply and removal, general animal health care, birth assistance) (aOR=3.73, 95%CI 2.00-6.94), lived as child on a ruminant farm (aOR= 2.24, 95%CI 1.17-4.28), presence of cat(s) in goat stable (aOR=2.54, 95%CI 1.24-5.21), distance residence to nearest stable ≤ 10 m (aOR=2.44, 95%CI 1.27-4.67), other goat breeds next to white dairy goat (aOR=3.38, 95%CI 1.61-7.09), combat other nuisance animals in 2008 via covering airspaces (aOR= 6.03, 95%CI 1.77-20.61), no farm boots for staff (aOR= 2.66, 95%CI 1.12-6.32), distance to nearest positive farm 0- <4 km (aOR=3.38, 95%CI 1.25-9.11)
Schønning [217]	Paddy workers	Leptospira spp.	Paddy fields near to home (OR=2.14, 95%CI 1.25-3.66), worked in a marshy land and/or having marshy lands near to home and/or work (OR= 2.26, 95%CI 1.35-3.79)
Şevketbeyoğlu[218]	Military forces	Chlamydia pneumoniae*	NA
Sharma[219]	Military forces	Hepatitis E virus*	Consumption of juice with ice from juice shops (aOR=15.7, 95% CI 8.3-29.3)

Shaw[220]	Abattoir workers (chicken)	Chlamydia psittaci*	NA
Shikova[221]	Female sex workers	Human papillomavirus (type 16, 31)	Age > 30 years versus age ≤ 20 years (OR=0.22, 95%CI 0.07-0.66), non-smokers versus smokers (OR=0.25, 95%CI 0.11-0.54)
Shimizu[222]	Live-poultry market workers	Avian Influenza A (H5N1)	None identified.
Steinberg[223]	Meat processing workers	SARS-CoV-2 virus*	NA
Sun[224]	Hotel workers	Legionella pneumophila	Legionella in cooling towers (OR=2.85, 95%CI 1.19-6.81), dissatisfaction with the indoor air quality (OR=2.05, 95%CI 1.13-3.71)
Sun[225]	Cattle farmers	Coxiella burnetii	Milking cattle (aOR=1.88, 95%CI 1.21-2.94), general healthcare of cattle (aOR= 2.40, 95%CI 1.46-3.93), birth assistance (aOR= 2.07, 95%CI 1.31-3.27), contact raw milk (aOR= 2.47, 95%CI 1.50-4.06), contact cattle manure (aOR=3.23, 95%CI 1.92-5.44), contact dead-born animals (aOR=3.45, 95%CI 2.16-5.50)
Sulaiman[226]	Gold miners	Morbillivirus Measles*	NA
Swai[227]	Abattoir workers	Brucella abortus	Slaughtering (aOR=5.74, 95%CI 1.25-25.22), cleaning (aOR=7.10, 95%CI 1.51-32.05)
Sweet[228]	Female sex workers	Human papilloma virus (type 35, 51 and 52)	HPV prevalence was notably higher in HIV-positive women (32.1%) compared to HIV-negative women (20.8%, p=0.03). The prevalence of high-risk HPV types was 27.4% in HIV-positive women and 18.2% in HIV-negative women (p=0.07).
Tabibi[229]	Animal breeders (cattle,	Hepatitis E virus, Leptospira	<u>Leptospira spp.:</u> cattle-

	swine)	spp., Coxiella burnetii, Borrelia burgdorferi(=), Brucella spp.(=), Salmonella spp.(=)	breeders (aOR=4.02, 95%CI 1.10,14.77)
Tan[230]	Military forces	Influenza A virus (H1N1pdm09), Influenza B virus, Coxsackievirus*	NA
Tang[231]	Male sex workers	HIV, Treponema pallidum	None identified.
Teague[232]	Military forces	Staphylococcus aureus*	NA
Texeira[233]	Swine workers	Hepatitis E virus	Having professions with exposure to pigs for more than 16.5 years (aOR=5.4, 95% CI 1.9-15.6)
Theamboonlers[234]	Military forces	Hepatitis A virus*	NA
Toepp[235]	Hunting dog caretakers	Borrelia burgdorferi	None identified.
Torén[236]	Welders	Streptococcus pneumoniae	Occupational exposure to fumes (aOR=1.11, 95%CI 1.01- 1.21), silica dust (aOR=1.33, 95%CI 1.11-1.58)
Torres-Gonzalez[237]	Dairy farm workers	Mycobacterium bovis	High exposure activity (direct contact with livestock in closed spaces) (aOR=6.09, 95%CI 2.04-18.23)
Toseva[238]	Wastewater treatment workers	Hepatitis A virus	Operators (OR=2.91, 95% CI 1.15-7.39), maintenance personnel (OR=4.3, 95% CI 1.08-17.17)
Traoré[239]	Butchers(pork)	Hepatitis E virus	None identified.
Tschopp[240]	Sewage workers	Hepatitis E virus(=), Helicobacter pylori(=)	None identified.
Vainio[241]	Military forces	Streptococcus pneumoniae*	None identified.
van Cleef[242]	Abattoir workers(pigs)	MRSA	Working with live pigs (OR= 38.2, 95%CI 6.3-∞)
van Cleef[243]	Field workers (pigs, veal calves)	MRSA	MRSA acquisition in relation to MRSA prevalence among farm animals (per 10% increase in prevalence): pigs (aOR= 2.04, 95%CI 1.24-3.34), veal (aOR=1.28, 95%CI 1.06-1.53)
van Veen[244]	Sex workers (female and male-to-female transgender)	HIV	Transgender sex work (aOR=22.9, 95%CI 6.5-80.3), ever injected drugs (aOR=31.1, 95%CI

			8.7-111.2), African ethnicity (aOR=19.0, 95% CI 3.2-111.8), South European ethnicity (aOR= 7.2, 95%CI 1.2-42.0)
Verhaegh-Haasnoot[245]	Male sex workers (internet escorts)	HIV, Chlamydia trachomatis, Neisseria gonorrhoeae, Treponema pallidum, Hepatitis B virus	For newly diagnosed STI: 4-49 sex partners in the past 6 months (aOR=1.30, 95%CI 1.05-1.61), age 25 years and older versus < 25 years (aOR=0.68, 95%CI 0.54–0.85), positive STI history in the past 2 years (aOR=1.53, 95%CI 1.25-1.88), known to be HIV positive (aOR=2.53, 95%CI 1.86-3.43)
Vilay[246]	Military forces	Plasmodium falciparum, Plasmodium vivax	Working duration in forest areas (years) (Mann-Whitney test, p=0.03)
Vorsters[247]	Female sex workers	Human papillomavirus (type 6, 16, 18, 31, 33, 35, 39, 45, 51, 52, 53, 56, 58, 59, 66, 67, 68)	Age <21 years (OR=10.3, 95% CI 5.0-21.2)
Vu[248]	Female sex workers	HIV, Treponema pallidum, HSV-2	Factors associated with HIV infections: age (continuous) (aOR=1.07, 95%CI 1.04-1.1), education (some Secondary versus some Primary) (aOR=0.6, 95%CI 0.4–0.9), personal income (TZS) from sex work (120,001-350,000 versus <120,001) (aOR=0.7, 95%CI 0.5-0.99), tested positive for HSV-2 (aOR=3.3, 95%CI 2.3-4.6)
Wadl[249]	Military forces	Norovirus*	Salad consumption on 6 January (aOR=8.1, 95%CI 1.5-45.4) and 7 January (aOR=15.7, 95%CI 2.2-74.1), prepared by infected canteen employee
Wang[250]	Poultry market workers	Avian influenza A(H7N9)	Female sex (aOR=2.71, 95% CI 1.1-6.71), ≥10 years of occupational

			exposure (aOR=3.59, 95% CI 1.25-10.35)
Wang[251]	Female sex workers	Human papillomavirus (type 16, 52, 58)	Age 31-35 years versus ≤ 25 years (aOR=0.4, 95%CI 0.17-0.91). Condom use: occasionally versus always (aOR=3.01, 95%CI 1.73-5.23), never versus always (aOR=4.65, 95%CI 2.04-10.57)
Watier-Grillot[252]	Military forces	Norovirus (genogroup II)*	Consumption of infected shrimp salad (aOR=2.6, 95%CI 1.2-6.0), pasta salad (aOR= 2.9, 95%CI 1.3-6.4), mashed potatoes (aOR= 2.4, 95%CI 1.0-5.4)
Wickersham[253]	Cisgender and Transgender Women Sex Workers	HIV, Treponema pallidum, Chlamydia trachomatis, Neisseria gonorrhoeae	None identified.
Wilczynski[254]	Firefighters	Cryptosporidium parvum*	Carrying or leading calves from the burning barn (Relative Risk=2.88, 95%CI 1.04-12.76)
Wilken[255]	Television crew	Coccidioides immitis*	NA
Williams[256]	Poultry processing plant workers	Chlamydia psittaci*	Worked in, or visited, killing or automated evisceration areas (aOR= 13.9, 95%CI 1.9-99.5). Risk of recent infection because worked in, or visited killing or automated evisceration areas, stratified for the use of eye protection (mOR=8.1, 95%CI 1.3-49.4). Risk of recent infection by touching face with hands contaminated with viscera or blood, stratified for the use of eye protection (mOR=5.0, 95%CI 1.08-22.9), and stratified for the use of respiratory protection with FFP3 mask (mOR= 6.5, 95%CI 1.4-30.1).
Wilson[257]	Slaughterhouse and	Coxiella burnetii*	Passed through the stores

	cutting Plant workers		(OR=3.2, 95%CI 1.7-6.3), passed through walkway 2 (OR=2.1, 95%CI 1.0-4.3), male gender (OR=5.2, 95%CI 1.1-24.0)
Wongsanuphat[258]	Factory workers	Measles morbillivirus*	NA
Wu[259]	Swine workers	Swine influenza virus (H1N1)	Age group 26-50 years (aOR=2.70, 95%CI 1.15-6.38)
Wu[260]	Poultry workers	Avian influenza virus (H9N2)	Wholesale live poultry markets (OR=6.4, 95%CI 1.8-22.8), retail live poultry markets (OR=9.9, 95%CI 2.4-40.9)
Yang[261]	Poultry workers	Influenza virus A(H7N9)*	NA
Ye[262]	Pig workers	MDRSA (CC9, IEC-negative, tetracycline-resistant), MRSA (CC9)	Carriage of MDRSA: frequency of pig occupational contact(hours per day) < 8 (aPR=2.51, 95%CI 1.26-4.98), ≥ 8 (aPR=4.41, 95%CI 2.62-7.41). Carriage of MRSA: frequency of pig occupational contact(hours per day) <8 (aPR=5.11, 95%CI 2.16-12.09), ≥ 8 (aPR=9.54, 95%CI 4.77–19.06)
Ye[263]	Livestock workers	MRSA (CC9)	Contact with pig (aOR= 6.58, 95%CI 3.50-12.38), contact with poultry (aOR=4.94, 95%CI 1.32-18.41), contact with other animal (aOR=4.50, 95%CI 0.88-22.98), frequency of livestock contact (h/d) (aOR=2.25, 95%CI 1.72-2.94), frequency of pig contact (h/d) (aOR=2.30, 95%CI 1.74-3.06), short-term duration of livestock contact (mo) (aOR=1.72, 95%CI 1.34-2.21), short-term duration of pig contact (mo) (aOR=1.75, 95%CI 1.34-2.28)

Yoon[264]	Military forces	Mycobacterium tuberculosis	Location (with the index cases): different floor in a same building (aOR=3.43, 95%CI 1.39-8.47), next room (aOR=5.19, 95%CI 2.34-11.52), same room aOR=10.93, 95%CI 4.93-24.25)
Younis[265]	Poultry farm workers	S. aureus, Candida albicans, Aspergillus flavus, Aspergillus fumigatus	None identified.
Yu[266]	Female sex workers	HIV, Hepatitis C virus	None identified.
Yu[267]	Military forces	Adenovirus type 7*	NA
Zákutná[268]	Agricultural and forestry workers	Borrelia burgdorferi	Men (OR=1.98, 95%CI 1.01-3.91), age > 30 years (OR=3.75, 95%CI 1.42-9.94)
Zalla[269]	Female sex workers	HIV, Treponema pallidum	None identified.
Zermiani[270]	Female sex workers	HIV, Hepatitis B virus, Hepatitis C virus (=), Treponema pallidum	Infection: age (aOR=1 .07, 95%CI 1.01-1.14), recent immigration year (aOR= 0.88, 95%CI 0.79-0.98)

*Outbreak or case series report; (=) No association found in the current study, NA=not applicable, aOR=adjusted Odds Ratio, mOR= Mantel-Haenszel Odds Ratio, aHR=adjusted Hazard Ratio, aPR= adjusted Prevalence Ratio, aRR=adjusted Relative Risk, aRisk ratio=adjusted Risk Ratio, IRR=Incidence Rate Ratio, STI: Sexually Transmitted Infections, MDRSA: Multidrug-Resistant S. aureus

References of the included articles

1. (2010). "Outbreak of 2009 pandemic influenza a (H1N1) on a Peruvian Navy ship - June-July 2009." Morb Mortal Wkly Rep 59(6): 162-165.
2. Abseno, M., et al. (2014). "Tuberculosis among Addis Ababa city bus drivers and cash collectors." ETHIOP MED J(SUPPL. 1): 31-35.
3. Adjemian, J., et al. (2011). "Outbreak of Marburg hemorrhagic fever among miners in kamwenge and ibanda Districts, Uganda, 2007." J INFECT DIS 204(SUPPL. 3): S796-S799.
4. Alavi, S. M. and M. M. Khoshkho (2014). "Seroprevalence study of leptospirosis among rice farmers in khuzestan province, South west iran, 2012." Jundishapur j. microbiology 7(7): e11536.
5. Allard, A., et al. (2013). "Histoplasmosis outbreak associated with the renovation of an old house - Quebec, Canada, 2013." Morb Mortal Wkly Rep 62(51-52): 1041-1044.
6. Alonso, E., et al. (2015). "Q Fever Outbreak among Workers at a Waste-Sorting Plant." PLoS ONE 10(9): e0138817.
7. Alonso, E., et al. (2019). "A Q fever outbreak associated to courier transport of pets." PLoS ONE 14(11): e0225605.
8. Al-Sayyad, A. S., et al. (2011). "Mumps outbreak among military and police training camps in Bahrain: Epidemiology and evaluation of control measures." Internet J Infect Dis 10(1): 5.

9. Al-Thaqafy, M. S., et al. (2013). "Hepatitis B virus among Saudi National Guard Personnel: Seroprevalence and risk of exposure." J Infect Public Health 6(4): 237-245.
10. Alvarado, G. R., et al. (2020). "Symptom Characterization and Outcomes of Sailors in Isolation after a COVID-19 Outbreak on a US Aircraft Carrier." JAMA Netw Open 3(10).
11. Alvarado-Esquivel, C., et al. (2015). "Leptospira Exposure and Waste Pickers: A Case-Control Seroprevalence Study in Durango, Mexico." J Clin Med Res 7(8): 637-640.
12. Alvarado-Esquivel, C., et al. (2016). "High Seroprevalence of Leptospira Exposure in Meat Workers in Northern Mexico: A Case-Control Study." J Clin Med Res 8(3): 231-236.
13. Alvarado-Esquivel, C., et al. (2011). "Toxoplasma gondii infection in workers occupationally exposed to raw meat." OCCUP MED 61(4): 265-269.
14. Alvarado-Esquivel, C., et al. (2014). "Lack of association between Toxoplasma gondii infection and occupational exposure to animals." Eur. j. microbiol. immunol. 4(4): 184-192.
15. Ambrose, J., et al. (2014). "Large outbreak of Legionnaires' disease and Pontiac fever at a military base." EPIDEMIOL INFECT 142(11): 2336-2346.
16. Amsalu, A., et al. (2016). "The exposure rate to hepatitis B and C viruses among medical waste handlers in three government hospitals, southern Ethiopia." Epidemiol Health 38: e2016001.
17. Andriopoulos, P., et al. (2018). "Brucella seroprevalence in a high-risk population in Greece: A cross-sectional study." Interdiscip Perspect Infect Dis 2018: e8751921.
18. Ansari-Moghaddam, A., et al. (2016). "The prevalence of Hepatitis B Virus among municipal solidwaste workers: Necessity for immunization of at-risk groups." Hepat Mon 16(3): e30887.
19. Aquino, T. L., et al. (2014). "Influenza outbreak in a vaccinated population--USS Ardent, February 2014." MMWR Morb Mortal Wkly Rep 63(42): 947-949.
20. Archer, B. N., et al. (2011). "Outbreak of Rift Valley fever affecting veterinarians and farmers in South Africa, 2008." S AFR MED J 101(4): 263-266.
21. Armstrong, P. A., et al. (2018). "Outbreak of Severe Histoplasmosis among Tunnel Workers - Dominican Republic, 2015." CLIN INFECT DIS 66(10): 1550-1557.
22. Awah-Ndukum, J., et al. (2018). "Seroprevalence and risk factors of brucellosis among slaughtered indigenous cattle, abattoir personnel and pregnant women in Ngaoundere, Cameroon." BMC Infect Dis 18(1): 611.
23. Awosanya, E. J., et al. (2013). "Factors associated with probable cluster of Leptospirosis among kennel workers in Abuja, Nigeria." Pan Afr Med J (16): 144.
24. Bailey, M. S., et al. (2012). "Outbreak of zoonotic cutaneous leishmaniasis with local dissemination in Balkh, Afghanistan." J R Army Med Corps 158(3): 225-228.
25. Bansal, M., et al. (2017). "Seroepidemiology and molecular characterization of hepatitis E virus infection in swine and occupationally exposed workers in Punjab, India." Zoonoses Public Health 64(8): 662-672.
26. Banta, J., et al. (2016). "Notes from the Field: Outbreak of Hand, Foot, and Mouth Disease Caused by Coxsackievirus A6 Among Basic Military Trainees - Texas, 2015." MMWR Morb Mortal Wkly Rep 65(26): 678-680.
27. Baral, S., et al. (2014). "Reconceptualizing the HIV epidemiology and prevention needs of female sex workers (FSW) in Swaziland." PLoS ONE 9(12): e115465.

28. Barnhart, D. A., et al. (2019). "Structural, interpersonal, psychosocial, and behavioral risk factors for HIV acquisition among female bar workers in Dar es Salaam, Tanzania." AIDS CARE PSYCHOL SOCIO-MED ASP AIDS HIV 31(9): 1096-1105.
29. Beaudoin, A., et al. (2010). "Serologic survey of swine workers for exposure to H2N3 swine influenza A." Influ Other Respir Viruses 4(3): 163-170.
30. Beheshti, S., et al. (2010). "Seroprevalence of brucellosis and risk factors related to high risk occupational groups in Kazeroon, South of Iran." Int J Occup Environ Med 1(2): 62-68.
31. Bellali, H., et al. (2017). "Zoonotic Cutaneous Leishmaniasis Prevalence Among Farmers in Central Tunisia, 2014." J Agromedicine 22(3): 244-250.
32. Bernier, A., et al. (2020). "HIV and other sexually transmitted infections among female sex workers in Moscow (Russia): prevalence and associated risk factors." Sex Transm Infect Mar (18) pii: sextrans-2019-054299.
33. Beste, L. A., et al. (2019). "Prevalence of Hepatitis B Virus Exposure in the Veterans Health Administration and Association With Military-related Risk Factors." Clin Gastroenterol Hepatol Aug (5): eS1542-3565.
34. Bilman, F. B., et al. (2017). "Epidemiological analysis using pulsed-field gel electrophoresis of Salmonella enteritidis outbreak in factory workers." Jundishapur J Microbiol 10(9): e14144.
35. Birku, T., et al. (2015). "Prevalence of hepatitis B and C viruses infection among military personnel at Bahir Dar Armed Forces General Hospital, Ethiopia." BMC Res Notes 8: 737.
36. Boost, M. V., et al. (2015). "Prevalence of carriage and characterisation of methicillin-resistant Staphylococcus aureus in slaughter pigs and personnel exposed to pork carcasses." Hong Kong Med J 21(6): S36-S40.
37. Borkenhagen, L. K., et al. (2019). "High Risk of Influenza Virus Infection Among Swine Workers: Examining a Dynamic Cohort in China." CLIN INFECT DIS XX(XX): 1–8.
38. Boscarino, J. A., et al. (2014). "Risk factors for hepatitis C infection among Vietnam era veterans versus nonveterans: results from the Chronic Hepatitis Cohort Study (CHeCS)." J Community Health 39(5): 914-921.
39. Boyce, S. C., et al. (2020). "HIV Infection and Risk Heightened Among Female Sex Workers Who Entered the Sex Trade as Adolescents in Guatemala." AIDS and Behavior April (10): 10.1007/s10461-020-02841-1.
40. Brinker, A. J. and D. L. Blazes (2017). "An outbreak of Leptospirosis among United States military personnel in Guam." Trop Dis Travel Med Vaccines 3(1): 1-4.
41. Brooks, J., et al. (2012). "No evidence of cross-species transmission of mouse retroviruses to animal workers exposed to mice." TRANSFUSION 52(2): 317-325.
42. Brosh-Nissimov, T., et al. (2018). "An Outbreak of Microsporum canis infection at a military base associated with stray cat exposure and person-to-person transmission." MYCOSES 61(7): 472-476.
43. Cárcamo, C. P., et al. (2012). "Prevalences of sexually transmitted infections in young adults and female sex workers in Peru: A national population-based survey." Lancet Infect Dis 12(10): 765-773.
44. Carpentier, A., et al. (2012). "High hepatitis E virus seroprevalence in forestry workers and in wild boars in France." J CLIN MICROBIOL 50(9): 2888-2893.

45. Caruso, C., et al. (2017). "Hepatitis E Virus: A Cross-Sectional Serological and Virological Study in Pigs and Humans at Zoonotic Risk within a High-Density Pig Farming Area." Transbound Emerg Dis 64(5): 1443-1453.
46. Cassir, N., et al. (2020). "Outbreak of pneumococcal pneumonia among shipyard workers in Marseille, France, January to February 2020." Eurosurveillance 25(11): 2000162.
47. Cavaretto, L., et al. (2018). "Epidemiological and molecular analysis of hepatitis B virus infection in manicurists in Central Brazil." J MED VIROL 90(2): 277-281.
48. Chabata, S. T., et al. (2019). "Changes Over Time in HIV Prevalence and Sexual Behaviour Among Young Female Sex-Workers in 14 Sites in Zimbabwe, 2013-2016." AIDS Behav 23(6):1494-1507.
49. Chapman, A. S., et al. (2011). "Norovirus outbreak associated with person-to-person transmission, U.S. Air Force Academy, July 2011." MSMR 18(11): 2-5.
50. Chaudhry, M., et al. (2020). "Avian influenza at animal-human interface: One-health challenge in live poultry retail stalls of Chakwal, Pakistan." Influ Other Respir Viruses 14(3):257-265.
51. Chaussade, H., et al. (2013). "Hepatitis E virus seroprevalence and risk factors for individuals in working contact with animals." J Clin Virol 58(3): 504-508.
52. Chen, M., et al. (2017). "An emm5 group A streptococcal outbreak among workers in a factory manufacturing telephone accessories." Front Microbiol 8(JUN): 1156.
53. Cheng, J., et al. (2016). "Epidemiology and transmission characteristics of human adenovirus type 7 caused acute respiratory disease outbreak in military trainees in east China." Am J Transl Res 8(5): 2331-2342.
54. Choi, M. J., et al. (2015). "Live Animal Markets in Minnesota: A Potential Source for Emergence of Novel Influenza A Viruses and Interspecies Transmission." CLIN INFECT DIS 61(9): 1355-1362.
55. Coman, A., et al. (2013). "Serological evidence for avian H9N2 influenza virus infections among Romanian agriculture workers." J Infect Public Health 6(6): 438-447.
56. Cook, E. A. J., et al. (2017). "Risk factors for leptospirosis seropositivity in slaughterhouse workers in western Kenya." OCCUP ENVIRON MED 74(5): 357-365.
57. Cook, E. A. J., et al. (2017). "The sero-epidemiology of Rift Valley fever in people in the Lake Victoria Basin of western Kenya." PLoS Negl Trop Dis 11(7): e0005731.
58. Cosby, M. T., et al. (2013). "Outbreak of H3N2 influenza at a US military base in Djibouti during the H1N1 pandemic of 2009." PLoS ONE 8(12): e82089.
59. Cowan, J. B. and T. S. Davis (2012). "Varicella outbreak among Afghan National Civil Order Police recruits-Herat Regional Military Training Center, Herat, Afghanistan, 2010." MIL MED 177(8): 924-927.
60. Crowell, T. A., et al. (2017). "Stigma, access to healthcare, and HIV risks among men who sell sex to men in Nigeria." J Int AIDS Soc 20(1): e 21489.
61. Crucitti, T., et al. (2010). "Trichomonas vaginalis is highly prevalent in adolescent girls, pregnant women, and commercial sex workers in Ndola, Zambia." SEX TRANSM DIS 37(4): 223-227.
62. Cui, W., et al. (2016). "Hepatitis E seroprevalence and related risk factors among seafood processing workers: A cross-sectional survey in Shandong Province, China." Int J Infect Dis 49: 62-66.
63. Cummings, K. C., et al. (2010). "Point-source outbreak of coccidioidomycosis in construction workers." EPIDEMIOL INFECT 138(4): 507-511.

64. Das, R., et al. (2012). "Occupational coccidioidomycosis in California: Outbreak investigation, respirator recommendations, and surveillance findings." J OCCUP ENVIRON MED 54(5): 564-571.
65. De Keukeleire, M., et al. (2016). "Individual and environmental factors associated with the seroprevalence of Borrelia burgdorferi in Belgian farmers and veterinarians." Infect Ecol Epidemiol 6: 32793.
66. De Keukeleire, M., et al. (2018). "Seroprevalence of Borrelia burgdorferi in Belgian forestry workers and associated risk factors." Parasit Vectors 11(1): 277.
67. De Lange, M. M. A., et al. (2014). "Coxiella burnetii seroprevalence and risk factors in sheep farmers and farm residents in the Netherlands." EPIDEMIOL INFECT 142(6): 1231-1244.
68. De Laval, F., et al. (2011). "Severe norovirus outbreak among soldiers in the field: Foodborne followed by person-to-person transmission." CLIN INFECT DIS 53(4): 399-400.
69. De Marco, M. A., et al. (2013). "Evidence of cross-reactive immunity to 2009 pandemic influenza A virus in workers seropositive to swine H1N1 influenza viruses circulating in Italy." PLoS ONE 8(2): e57576.
70. De Souza, R. L., et al. (2020). "Prevalence of syphilis in female sex workers in three countryside cities of the state of Pará, Brazilian Amazon." BMC Infect Dis 20(1): 129.
71. Delgado Naranjo, J., et al. (2011). "Study and management of a Q fever outbreak among machine tool workers in the basque country (Spain)." Epidemiol Res Intern 2011: e136946.
72. Di Renzi, S., et al. (2010). "Risk of acquiring tick-borne infections in forestry workers from Lazio, Italy." EUR J CLIN MICROBIOL INFECT DIS 29(12): 1579-1581.
73. Diabate, S., et al. (2018). "Gonorrhea, Chlamydia and HIV incidence among female sex workers in Cotonou, Benin: A longitudinal study." PLoS ONE 13(5): e0197251.
74. Dongliu, Y., et al. (2016). "Outbreak of acute febrile respiratory illness caused by human adenovirus B P14H11F14 in a military training camp in Shandong China." Arch Virol 161(9): 2481-2489.
75. Dreyfus, A., et al. (2014). "Sero-prevalence and risk factors for leptospirosis in abattoir workers in New Zealand." Int J Environ Res Public Health 11(2): 1756-1775.
76. Dreyfus, A., et al. (2015). "Risk of infection and associated influenza-like disease among abattoir workers due to two Leptospira species." EPIDEMIOL INFECT 143(10): 2095-2105.
77. Duggan, J. M., et al. (2017). "A seroprevalence study to determine the frequency of hantavirus infection in people exposed to wild and pet fancy rats in England." EPIDEMIOL INFECT 145(12): 2458-2465.
78. Eassa, S. M., et al. (2016). "Risk Factors Associated with Parasitic Infection among Municipality Solid-Waste Workers in an Egyptian Community." J PARASITOL 102(2): 214-221.
79. Esmaeili, S., et al. (2019). "Seroepidemiological study of Q fever, brucellosis and tularemia in butchers and slaughterhouses workers in Lorestan, western of Iran." Comp Immunol Microbiol Infect Dis 66: e101322.
80. Ferreira-Junior, O. D. C., et al. (2018). "Prevalence estimates of HIV, syphilis, hepatitis B and C among female sex workers (FSW) in Brazil, 2016." MEDICINE 97(1S): S3-S8.
81. Fragaszy, E., et al. (2016). "Increased risk of A(H1N1)pdm09 influenza infection in UK pig industry workers compared to a general population cohort." Influ Other Respir Viruses 10(4): 291-300.

82. Geenen, P. L., et al. (2013). "Prevalence of livestock-associated MRSA on Dutch broiler farms and in people living and/or working on these farms." EPIDEMIOL INFECT 141(5): 1099-1108.
83. Geng, Y., et al. (2019). "High seroprevalence of hepatitis E virus in rabbit slaughterhouse workers." Transboundary Emer Dis 66: 1085–1089.
84. Gobet, A., et al. (2014). "Mumps among highly vaccinated people: Investigation of an outbreak in a French Military Parachuting Unit, 2013." J INFECT 68(1): 101-102.
85. Goldstein, R. R., et al. (2017). "Higher prevalence of coagulase-negative staphylococci carriage among reclaimed water spray irrigators." Sci Total Environ 595: 35-40.
86. González, C., et al. (2011). "Higher incidence and persistence of high-risk human papillomavirus infection in female sex workers compared with women attending family planning." Int J Infect Dis 15(10): e688-e694.
87. Govender, N. P., et al. (2015). "An Outbreak of Lymphocutaneous Sporotrichosis among Mine-Workers in South Africa." PLoS Negl Trop Dis 9(9): e0004096.
88. Guerra, R. I., et al. (2019). "A cluster of the first reported Plasmodium ovale spp. infections in Peru occuring among returning un peace-keepers, a review of epidemiology, prevention and diagnostic challenges in nonendemic regions." Malar J 18(1): 176.
89. Gutierrez Garitano, I., et al. (2011). "Shigellosis outbreak linked to canteen-food consumption in a public institution: a matched case-control study." EPIDEMIOL INFECT 139(12): 1956-1964.
90. Hargreaves, J. R., et al. (2016). "Cohort analysis of program data to estimate HIV incidence and uptake of HIV-related services among female sex workers in Zimbabwe, 2009-2014." J ACQUIRED IMMUNE DEFIC SYNDR 72(1): e1-e8.
91. Harris, P. N. A., et al. (2016). "An outbreak of scrub typhus in military personnel despite protocols for antibiotic prophylaxis: doxycycline resistance excluded by a quantitative PCR-based susceptibility assay." Microbes Infect 18(6): 406-411.
92. Hatcher, S. M., et al. (2017). "The prevalence of antibiotic-resistant Staphylococcus aureus nasal carriage among industrial hog operation workers, community residents, and children living in their households: North Carolina, USA." ENVIRON HEALTH PERSPECT 125(4): 560-569.
93. Hensen, B., et al. (2019). "HIV risk among young women who sell sex by whether they identify as sex workers: analysis of respondent-driven sampling surveys, Zimbabwe, 2017." J Int AIDS Soc 22(12).
94. Hoan, N. X., et al. (2019). "High Hepatitis e virus (HEV) Positivity among Domestic Pigs and Risk of HEV Infection of Individuals Occupationally Exposed to Pigs and Pork Meat in Hanoi, Vietnam." Open Forum Infect Dis 6(9): 1-7.
95. Hinjoy, S., et al. (2013). "A cross-sectional study of hepatitis E virus infection in healthy people directly exposed and unexposed to pigs in a rural community in northern Thailand." Zoonoses Public Health 60(8): 555-562.
96. Ho, Z. J. M., et al. (2015). "Emergence of norovirus GI.2 outbreaks in military camps in Singapore." Int J Infect Dis 31: e23-e30.
97. Huang, S. Y., et al. (2015). "Serological comparison of antibodies to avian influenza viruses, subtypes H5N2, H6N1, H7N3 and H7N9 between poultry workers and non-poultry workers in Taiwan in 2012." EPIDEMIOL INFECT 143(14): 2965-2974.

98. Huang, Y., et al. (2016). "Changes in prevalence of HIV or syphilis among male sex workers and non-commercial men who have sex with men in Shenzhen, China: Results of a second survey." PLoS ONE 11(12).
99. Huijbers, P. M. C., et al. (2014). "Extended-spectrum and AmpC β-lactamase-producing Escherichia coli in broilers and people living and/or working on broiler farms: Prevalence, risk factors and molecular characteristics." J Antimicrob Chemother 69(10): 2669-2675.
100. Hulin, V., et al. (2015). "Host preference and zoonotic potential of Chlamydia psittaci and C. gallinacea in poultry." Pathogens Dis 73(1): 1-11.
101. Hurtado, A., et al. (2017). "Environmental sampling coupled with real-time PCR and genotyping to investigate the source of a Q fever outbreak in a work setting." EPIDEMIOL INFECT 145(9): 1834-1842.
102. Hwang, S. M., et al. (2013). "Outbreak of febrile respiratory illness caused by adenovirus at a south Korean military training facility: Clinical and radiological characteristics of adenovirus pneumonia." Jpn J Infect Dis 66(5): 359-365.
103. Iser, B. P. M., et al. (2012). "Outbreak of Neisseria meningitidis C in workers at a large food-processing plant in Brazil: Challenges of controlling disease spread to the larger community." EPIDEMIOL INFECT 140(5): 906-915.
104. Isler, M. A., et al. (2013). "Screening employees of services for homeless individuals in Montreal for tuberculosis infection." J Infect Public Health 6(3): 209-215.
105. Ismail, H., et al. (2020). "An outbreak of cutaneous abscesses caused by Panton-Valentine leukocidin-producing methicillin-susceptible Staphylococcus aureus among gold mine workers, South Africa, November 2017 to March 2018." BMC Infect Dis 20(1).
106. Jahfari, S., et al. (2014). "High seroprevalence of Borrelia miyamotoi antibodies in forestry workers and individuals suspected of human granulocytic anaplasmosis in the Netherlands." New Microbes New Infect 2(5): 144-149.
107. Jeger, V., et al. (2011). "H1N1 outbreak in a Swiss military boot camp--observations and suggestions." Swiss Med Wkly 141: w13307.
108. Jia, H., et al. (2015). "Human papillomavirus infection and cervical dysplasia in female sex workers in Northeast China: an observational study." BMC Public Health 15: 695.
109. Jurke, A., et al. (2015). "Serological survey of Bartonella spp., Borrelia burgdorferi, Brucella spp., Coxiella burnetii, Francisella tularensis, Leptospira spp., Echinococcus, Hanta-, TBE- and XMR-virus infection in employees of two forestry enterprises in North Rhine-Westphalia, Germany, 2011-2013." Int J Med Microbiol 305(7): 652-662.
110. Kajon, A. E., et al. (2010). "Outbreak of Febrile Respiratory Illness Associated with Adenovirus 11a Infection in a Singapore Military Training Camp." J CLIN MICROBIOL 48(4): 1438-1441.
111. Kang, Y. H., et al. (2017). "Hepatitis E virus seroprevalence among farmers, veterinarians and control subjects in Jilin province, Shandong province and Inner Mongolia Autonomous Region, China." J MED VIROL 89(5): 872-877.
112. Katelaris, A. L., et al. (2019). "Investigation and response to an outbreak of leptospirosis among raspberry workers in Australia, 2018." Zoonoses Public Health 67(1): 35-43.

113. Kato, H., et al. (2020). "Rubella outbreak among workers in three small- and medium-size business establishments associated with imported genotype 1E rubella virus—Shizuoka, Japan, 2015." VACCINE 38(46): 7278-7283.
114. Kayali, G., et al. (2011). "Evidence of infection with H4 and H11 avian influenza viruses among Lebanese chicken growers." PLoS ONE 6(10): e26818.
115. Kayali, G., et al. (2010). "Evidence of previous avian influenza infection among US Turkey workers." Zoonoses Public Health 57(4): 265-272.
116. Kayali, G., et al. (2011). "Serologic evidence of Avian metapneumovirus infection among adults occupationally exposed to Turkeys." Vector Borne Zoonotic Dis 11(11): 1453-1458.
117. Kho, K. L., et al. (2017). "Rickettsial seropositivity in the indigenous community and animal farm workers, and vector surveillance in Peninsular Malaysia." Emerg Microbes Infect 6(4): e18.
118. Khounvisith, V., et al. (2018). "High circulation of Hepatitis E virus in pigs and professionals exposed to pigs in Laos." Zoonoses Public Health 65(8): 1020-1026.
119. Khurelbaatar N, et al. (2014) Little Evidence of Avian or Equine Influenza Virus Infection among a Cohort of Mongolian Adults with Animal Exposures, 2010–2011. PLoS ONE 9(1): e85616.
120. Kissling, E., et al. (2012). "B. anthracis in a wool-processing factory: Seroprevalence and occupational risk." EPIDEMIOL INFECT 140(5): 879-886.
121. Klumb, C. A., et al. (2020). "Animal agriculture exposures among Minnesota residents with zoonotic enteric infections, 2012-2016." EPIDEMIOL INFECT 148: e55.
122. Knust, B., et al. (2014). "Lymphocytic choriomeningitis virus in employees and mice at multipremises feeder-rodent operation, United States, 2012." Emerg Infect Dis 20(2): 240-247.
123. Kobayashi, A., et al. (2020). "Epidemiology of a workplace measles outbreak dominated by modified measles cases at Kansai international airport, Japan, during august–september 2016." VACCINE 38(32): 4996-5001.
124. Krueger, W. S., et al. (2014). "No evidence for zoonotic transmission of H3N8 canine influenza virus among US adults occupationally exposed to dogs." Influ Other Respir Viruses 8(1): 99-106.
125. Krueger, W. S., et al. (2014). "Evidence for Unapparent Brucella canis Infections among Adults with Occupational Exposure to Dogs." Zoonoses Public Health 61(7): 509-518.
126. Krumbholz, A., et al. (2010). "Prevalence of antibodies to swine influenza viruses in humans with occupational exposure to pigs, Thuringia, Germany, 2008-2009." J MED VIROL 82(9): 1617-1625.
127. Krumbholz, A., et al. (2012). "Prevalence of hepatitis E virus-specific antibodies in humans with occupational exposure to pigs." Med Microbiol Immunol 201(2): 239-244.
128. Kunwar, C. R. and R. Prakash (2015). "Dengue outbreak in a large military station: Have we learnt any lesson?" Med J Armed Forces India 71(1): 11-14.
129. Kupferman, T., et al. (2017). "Case Report: A Cluster of Three Leptospirosis Cases in a New York City Abattoir and an Unusual Complication in the Index Case." AM J TROP MED HYG 97(6): 1679-1681.
130. Lagler, H., et al. (2014). "Hepatitis E virus seroprevalence in austrian adults: A nationwide cross-sectional study among civilians and military professionals." PLoS ONE 9(2): e87669.

131. Lan F.Y., et al. (2020). "Association between SARS-CoV-2 infection, exposure risk and mental health among a cohort of essential retail workers in the USA". Occup Environ Med. 2020 Oct 30:oemed-2020-106774.
132. Laroucau, K., et al. (2015). "Outbreak of psittacosis in a group of women exposed to Chlamydia psittaci-infected chickens." Euro Surveill 20(24): e21155.
133. Larson, K. R., et al. (2015). "Serological evidence of equine influenza infections among persons with horse exposure, Iowa." J Clin Virol 67: 78-83.
134. Lee, J. T., et al. (2013). "Seroprevalence of Hepatitis E Virus Infection among Swine Farmers and the General Population in Rural Taiwan." PLoS ONE 8(6): e67180.
135. Lee, V. J., et al. (2009). "An outbreak of salmonella gastrointestinal illness in a military camp." Ann Acad Med Singapore 38(3): 207-211.
136. Li, L., et al. (2017). "Nasal carriage of methicillin-resistant coagulase-negative staphylococci in healthy humans is associated with occupational pig contact in a dose-response manner." Vet Microbiol 208: 231-238.
137. Li, S., et al. (2016). "Avian influenza virus H9N2 seroprevalence and risk factors for infection in occupational poultry-exposed workers in Tai'an of China." J MED VIROL 88(8): 1453-1456.
138. Li, Y., et al. (2013). "An outbreak of norovirus gastroenteritis associated with a secondary water supply system in a factory in south China." BMC Public Health 13: 283.
139. Li, Z., et al. (2014). "Seroprevalence of antibodies against SFTS virus infection in farmers and animals, Jiangsu, China." J Clin Virol 60(3): 185-189.
140. Liang, H., et al. (2014). "The prevalence of hepatitis e virus infections among swine, swine farmers and the general population in Guangdong Province, China." PLoS ONE 9(2): e88106.
141. Linkevicius, M., et al. (2019). "Outbreak of invasive pneumococcal disease among shipyard workers, Turku, Finland, May to November 2019." Eurosurveillance 24(49): 1900681.
142. Liu, P. Y., et al. (2009). "Outbreak of influenza A and B among military recruits: Evidence from viral culture and polymerase chain reaction." J Microbiol Immunol Infect 42(2): 114-121.
143. Liu, W., et al. (2019). "Localized Outbreaks of Epidemic Polyarthritis among Military Personnel Caused by Different Sublineages of Ross River Virus, Northeastern Australia, 2016-2017." Emerging Infect Dis 25(10): 1793-1801.
144. Lopes-João, A., et al. (2015). "Multiple enteropathogenic viruses in a gastroenteritis outbreak in a military exercise of the Portuguese Army." J Clin Virol 68: 73-75.
145. López-Robles, G., et al. (2012). "Seroprevalence and Risk Factors for Swine Influenza Zoonotic Transmission in Swine Workers from Northwestern Mexico." Transboundary Emer Dis 59(2): 183-188.
146. Lord, H., et al. (2016). "A Q fever cluster among workers at an abattoir in south-western Sydney, Australia, 2015." Western Pac Surveill Response J 7(4): 21-27.
147. Ma, M., et al. (2015). "Serological evidence and risk factors for swine influenza infections among Chinese swine workers in guangdong province." PLoS ONE 10(5): e0128479..
148. Macdonald, E., et al. (2016). "National outbreak of Yersinia enterocolitica infections in military and civilian populations associated with consumption of mixed salad, Norway, 2014." Euro Surveill 21(34): e30321.

149. Marinho, T. A., et al. (2014). "Epidemiology of hepatitis B virus infection among recyclable waste collectors in central Brazil." Rev Soc Bras Med Trop 47(1): 18-23.
150. Marinho, T. A., et al. (2013). "Prevalence of hepatitis C virus infection among recyclable waste collectors in Central-West Brazil." Mem Inst Oswaldo Cruz 108(4): 519-522.
151. Marra, E., et al. (2018). "Vaginal and anal human papillomavirus infection and seropositivity among female sex workers in Amsterdam, the Netherlands: Prevalence, concordance and risk factors." J INFECT 76(4): 393-405.
152. Martins, R. M. B., et al. (2014). "Seroprevalence of hepatitis E antibodies in a population of recyclable waste pickers in Brazil." J Clin Virol 59(3): 188-191.
153. Matos, M. A., et al. (2017). "Viral hepatitis in female sex workers using the Respondent-Driven Sampling." Rev Saude Publica 51: 65.
154. Mayet, A., et al. (2011). "Food-borne outbreak of norovirus infection in a French military parachuting unit, april 2011." Euros Surveill 16(30): e19930.
155. Mayet, A., et al. (2011). "Novel influenza A(H1N1) outbreak among French armed forces in 2009: Results of Military Influenza Surveillance System." PUBLIC HEALTH 125(8): 494-500.
156. Mayet, A., et al. (2013). "The measles outbreak in the French military forces - 2010-2011: Results of epidemiological surveillance." J INFECT 66(3): 271-277.
157. Mbareche, H., et al. (2019). "Bioaerosols Play a Major Role in the Nasopharyngeal Microbiota Content in Agricultural Environment." Int J Environ Res Public Health 16: e1375.
158. McCurdy, S. A., et al. (2020). "Risk for coccidioidomycosis among hispanic farm workers, California, USA, 2018." Emerg Infect Dis 26(7): 1430-1437.
159. McKinnon, L. R., et al. (2014). "High HIV risk in a cohort of male sex workers from Nairobi, Kenya." Sex Transm Infect 90(3): 237-242.
160. McKinnon, L. R., et al. (2015). "Risk Factors for HIV Acquisition in a Prospective Nairobi-Based Female Sex Worker Cohort." AIDS Behav 19(12): 2204-2213.
161. Méndez, C., et al. (2019). "Prevalence of Trypanosoma cruzi infection in active military population of the Colombian National Army gathered in five departments." PLoS ONE 14(10): e0223611.
162. Mirzazadeh, A., et al. (2020). "Declining trends in HIV and other sexually transmitted infections among female sex workers in Iran could be attributable to reduced drug injection: A cross-sectional study." Sex Transm Infect 96(1): 68-75.
163. Moayedi-Nia, S., et al. (2016). "HIV, HCV, HBV, HSV, and syphilis prevalence among female sex workers in Tehran, Iran, by using respondent-driven sampling." AIDS CARE PSYCHOL SOCIO-MED ASP AIDS HIV 28(4): 487-490.
164. Mohd Ridzuan, J., et al. (2016). "Work environment-related risk factors for leptospirosis among plantation workers in tropical countries: Evidence from Malaysia." Int J Occup Environ Med 7(3): 156-163.
165. Mol, M. P. G., et al. (2016). "Seroprevalence of hepatitis B and C among domestic and healthcare waste handlers in Belo Horizonte, Brazil." WASTE MANAGE RES 34(9): 875-883.
166. Montuori, P., et al. (2009). "Wastewater workers and hepatitis A virus infection." Occup Med (Oxf) 59(7): 506-508.

167. Mor, Z. and M. Dan (2012). "Knowledge, attitudes, sexual practices and STI/HIV prevalence in male sex workers and other men who have sex in Tel Aviv, Israel: A cross-sectional study." Sex Transm Infect 88(8): 574-580.
168. Mossong, J., et al. (2009). "Mumps outbreak among the military in Luxembourg in 2008: epidemiology and evaluation of control measures." Euro Surveill 14(7): e19121.
169. Mostafavi, E., et al. (2019). "Seroprevalence of Q fever among high-risk occupations in the Ilam province, the west of Iran." PLoS ONE 14(2): e0211781.
170. Mughini-Gras, L., et al. (2017). "Hepatitis E virus infection in North Italy: High seroprevalence in swine herds and increased risk for swine workers." EPIDEMIOL INFECT 145(16): 3375-3384.
171. Mulders, M. N., et al. (2010). "Prevalence of livestock-associated MRSA in broiler flocks and risk factors for slaughterhouse personnel in the Netherlands." EPIDEMIOL INFECT 138(5): 743-755.
172. Munseri, P. J., et al. (2013). "Declining HIV-1 prevalence and incidence among Police Officers - a potential cohort for HIV vaccine trials, in Dar es Salaam, Tanzania." BMC Public Health 13: 722.
173. Muraguri, N., et al. (2015). "HIV and STI prevalence and risk factors among male sex workers and other men who have sex with men in nairobi, kenya." J ACQUIRED IMMUNE DEFIC SYNDR 68(1): 91-96.
174. Musyoki, H., et al. (2015). "Prevalence of HIV, sexually transmitted infections, and risk behaviours among female sex workers in Nairobi, Kenya: results of a respondent driven sampling study." AIDS Behav 19: S46-S58.
175. Mutagoma, M., et al. (2017). "Syphilis and HIV prevalence and associated factors to their co-infection, hepatitis B and hepatitis C viruses prevalence among female sex workers in Rwanda." BMC Infect Dis 17(1): 525.
176. Narayanan, P., et al. (2013). "An exploration of elevated HIV and STI risk among male sex workers from India." BMC Public Health 13: 1059.
177. National Institute for Infectious Diseases, Shinjuku-ku, Tokyo, Japan (2020). “Epidemiology of COVID-19 Outbreak on Cruise Ship Quarantined at Yokohama, Japan, February 2020”. Emerg Infect Dis 26(11):2591-2597.
178. Navdarashvili, A., et al. (2016). "Human anthrax outbreak associated with livestock exposure: Georgia, 2012." EPIDEMIOL INFECT 144(1): 76-87.
179. Neo, F. J. X., et al. (2017). "Outbreak of caliciviruses in the Singapore military, 2015." BMC Infect Dis 17(1): 719.
180. Ngoupa, J. B., et al. (2019). "Seroprevalence and associated risk factors for Hepatitis B virus infection among barbers and their clients in two cities in Cameroon." South Afr J Infect Dis.
181. Nguyen, V. T., et al. (2019). "Limited contribution of non-intensive chicken farming to ESBL-producing Escherichia coli colonization in humans in Vietnam: an epidemiological and genomic analysis." J Antimicrob Chemother 74(3): 561-570.
182. Nicas, M. (2018). "A point-source outbreak of Coccidioidomycosis among a highway construction crew." J Occup Environ Hyg 15(1): 57-62.
183. Nielsen, L. E., et al. (2019). "Mumps outbreak and MMR IgG surveillance as a predictor for immunity in military trainees." VACCINE 37(42): 6139-6143.
184. Nielsen, S. Y., et al. (2013). "Prevalence of Coxiella burnetii in women exposed to livestock animals, Denmark, 1996 to 2002." Euro Surveill 18(28): e20528.

185. Nivesvivat, T., et al. (2016). "Methicillin-susceptible Staphylococcus aureus skin infections among military conscripts undergoing basic training in Bangkok, Thailand, in 2014." BMC Res Notes 9: 179.
186. Okoye, J., et al. (2013). "Serologic evidence of avian influenza virus infections among Nigerian agricultural workers." J MED VIROL 85(4): 670-676.
187. Oliveira-Filho, A. B., et al. (2019). "Hepatitis C virus among female sex workers: A cross-sectional study conducted along rivers and highways in the Amazon region." Pathogens 8(4): 236.
188. Oré, M., et al. (2015). "Outbreak of cutaneous leishmaniasis in peruvian military personnel undertaking training activities in the amazon basin, 2010." AM J TROP MED HYG 93(2): 340-346.
189. Pang, J., et al. (2015). "Risk factors for febrile respiratory illness and mono-viral infections in a semi-closed military environment: a case-control study." BMC Infect Dis 15: 288.
190. Park, J. N., et al. (2019). "Incidence and Predictors of Chlamydia, Gonorrhea and Trichomonas among a Prospective Cohort of Cisgender Female Sex Workers in Baltimore, Maryland." SEX TRANSM DIS 46 (12): 788-794.
191. Park, W. J., et al. (2015). "Respiratory syncytial virus outbreak in the basic military training camp of the republic of Korea Air Force." J Prev Med Public Health 48(1): 10-17.
192. Parveen, S. M. A., et al. (2016). "Leptospirosis seroprevalence among blue metal mine workers of Tamil Nadu, India." AM J TROP MED HYG 95(1): 38-42.
193. Patterson, T. L., et al. (2019). "Prevalence of HIV/STIs and correlates with municipal characteristics among female sex workers in 13 Mexican cities." Salud Publica Mex 61(2): 116-124.
194. Pawar, S. D., et al. (2012). "Avian influenza H9N2 seroprevalence among poultry workers in Pune, India, 2010." PLoS ONE 7(5): e36374.
195. Pirnay, J. P., et al. (2020). "Study of a SARS-CoV-2 Outbreak in a Belgian Military Education and Training Center in Maradi, Niger." Viruses 12(9).
196. Quan, C., et al. (2019). "Avian influenza A viruses among occupationally exposed populations, China, 2014-2016." Emerg Infect Dis 25(12): 2215-2225.
197. Rachiotis, G., et al. (2012). "Hepatitis A virus infection and the waste handling industry: A seroprevalence study." Int J Environ Res Public Health 9(12): 4498-4503.
198. Ramos, M., et al. (2013). "Outbreak of Group A beta hemolytic Streptococcus pharyngitis in a Peruvian military facility, April 2012." MSMR 20(6): 14-17.
199. Raza, N., et al. (2009). "Risk factors for scabies among male soldiers in Pakistan: Case-control study." East Mediterr Health J 15(5): 1105-1110.
200. Reeves, W. K., et al. (2015). "Case series: Chikungunya and dengue at a forward operating location." MSMR 22(5): 9-10.
201. Remoli, M. E., et al. (2018). "Seroprevalence survey of arboviruses in workers from Tuscany, Italy." MED LAV 109(2): 125-131.
202. Ricci, M. L., et al. (2010). "A preliminary assessment of the occupational risk of acquiring Legionnaires' disease for people working in telephone manholes, a new workplace environment for Legionella growth." AM J INFECT CONTROL 38(7): 540-545.

203. Richter, A., et al. (2012). "Prevalence of types of methicillin-resistant Staphylococcus aureus in turkey flocks and personnel attending the animals." EPIDEMIOL INFECT 140(12): 2223-2232.
204. Ringshausen, F. C., et al. (2013). "Frequent detection of latent tuberculosis infection among aged underground hard coal miners in the absence of recent tuberculosis exposure." PLoS ONE 8(12): e82005.
205. Rodkvamtook, W., et al. (2011). "Isolation and characterization of Orientia tsutsugamushi from rodents captured following a scrub typhus outbreak at a military training base, Bothong district, Chonburi province, central Thailand." AM J TROP MED HYG 84(4): 599-607.
206. Rosenberg Goldstein, R. E., et al. (2014). "Occupational exposure to Staphylococcus aureus and Enterococcus spp. among spray irrigation workers using reclaimed water." Int J Environ Res Public Health 11(4): 4340-4355.
207. Rossow, H., et al (2014). “Risk factors for pneumonic and ulceroglandular tularemia in Finland: A population-based case-control study”. Epidemiol Infect 142:2207-2216.
208. Royal, J., et al. (2013). "Seroepidemiologic survey for Coxiella burnetii among US military personnel deployed to southwest and central Asia in 2005." AM J TROP MED HYG 89(5): 991-995.
209. Sahibzada, S., et al. (2018). "Emergence of highly prevalent CA-MRSA ST93 as an occupational risk in people working on a pig farm in Australia." PLoS ONE 13(5): e0195510.
210. Sahneh, E., et al. (2019). "Investigation of Risk Factors Associated with Leptospirosis in the North of Iran (2011-2017)." J. res. health sci. 19(2): e00449.
211. Sakamoto, R., et al. (2009). "Is driving a car a risk for Legionnaires' disease?" EPIDEMIOL INFECT 137(11): 1615-1622.
212. Sanchez, M. A., et al. (2017). "Norovirus GII.17 Outbreak Linked to an Infected Post-Symptomatic Food Worker in a French Military Unit Located in France." Food Environ Virol 9(2): 234-237.
213. Sánchez-Anguiano, L. F., et al. (2019). "Prevalence of Chlamydia trachomatis infection diagnosed by polymerase chain reaction in female sex workers in a northern Mexican City." Eur J Microbiol Immunol 9(1): 5-8.
214. Santos, L., et al. (2013). "Acute histoplasmosis in three Mexican sewer workers." OCCUP MED 63(1): 77-79.
215. Sargianou, M., et al. (2013). "Crimean-Congo hemorrhagic fever: Seroprevalence and risk factors among humans in Achaia, western Greece." Int J Infect Dis 17(12): e1160-e1165.
216. Schimmer, B., et al. (2012). "Seroprevalence and risk factors for Coxiella burnetii (Q fever) seropositivity in dairy goat farmers' households in the Netherlands, 2009-2010." PLoS ONE 7(7): e42364.
217. Schønning, M. H., et al. (2019). "A Case-Control Study of Environmental and Occupational Risks of Leptospirosis in Sri Lanka." EcoHealth 16, 534–543.
218. Şevketbeyoğlu, H., et al. (2012). "Outbreak of chlamydia pneumoniae infection in a military unit: The distribution of lobar and segmental infiltration." Nobel Med 8(2): 26-31.
219. Sharma, S., et al. (2019). "Epidemiological investigation of viral hepatitis E outbreak in two colocated military training centers, North India, April–June 2016." Med J Armed Forces India 2017: 1-7.

220. Shaw, K. A., et al. (2019). "Psittacosis Outbreak among Workers at Chicken Slaughter Plants, Virginia and Georgia, USA, 2018." Emerging Infect Dis 25(11): 2143-2145.
221. Shikova, E., et al. (2011). "Prevalence of human papillomavirus infection among female sex workers in Bulgaria." INT J STD AIDS 22(5): 278-280.
222. Shimizu, K., et al. (2016). "Seroevidence for a high prevalence of subclinical infection with avian influenza a(h5n1) virus among workers in a live-poultry market in Indonesia." J INFECT DIS 214(12): 1929-1936.
223. Steinberg, J., et al. (2020). "COVID-19 Outbreak Among Employees at a Meat Processing Facility - South Dakota, March-April 2020." MMWR Morb Mortal Wkly Rep 69(31): 1015-1019.
224. Sun, H., et al. (2012). "Cooling towers contribute to the high seroprevalence of Legionella pneumophila antibody among hotel workers." J Public Health 20(4): 425-430.
225. Sun, W. W., et al. (2016). "Coxiella burnetii Seroprevalence and Risk Factors in Cattle Farmers and Farm Residents in Three Northeastern Provinces and Inner Mongolia Autonomous Region, China." BioMed Res Int 2016: e7059196.
226. Sulaiman, A. A., et al. (2020). "An outbreak of measles in gold miners in River Nile State, Sudan, 2011." East Mediterr Health J 26(2): 152-160.
227. Swai, E. S. and L. Schoonman (2009). "Human brucellosis: Seroprevalence and risk factors related to high risk occupational groups in Tanga municipality, Tanzania." Zoonoses Public Health 56(4): 183-187.
228. Sweet, K., et al. (2020). "Prevalence, incidence, and distribution of human papillomavirus types in female sex workers in Kenya." INT J STD AIDS 31(2): 109-118.
229. Tabibi, R., et al. (2013). "Occupational exposure to zoonotic agents among agricultural workers in Lombardy Region, Northern Italy." Ann Agric Environ Med 20(4): 676-681.
230. Tan, X. Q., et al. (2014). "Respiratory viral pathogens among Singapore military servicemen 2009 - 2012: Epidemiology and clinical characteristics." BMC Infect Dis 14(1): 204.
231. Tang, W., et al. (2015). "Burden of HIV and syphilis: A comparative evaluation between male sex workers and non-sex-worker men who have sex with men in urban China." PLoS ONE 10(5): e0126604.
232. Teague, N. S., et al. (2013). "Outbreak of staphylococcal food poisoning from a military unit lunch party - United States, July 2012." Morb Mortal Wkly Rep 62(50): 1026-1028.
233. Teixeira, J., et al. (2017). "Prevalence of hepatitis E virus antibodies in workers occupationally exposed to swine in Portugal." Med Microbiol Immunol 206(1): 77-81.
234. Theamboonlers, A., et al. (2009). "Molecular characterization of Hepatitis A Virus causing an outbreak among Thai navy recruits." Tropical Biomed 26(3): 352-359.
235. Toepp, A. J., et al. (2018). "Frequent Exposure to Many Hunting Dogs Significantly Increases Tick Exposure." Vector Borne Zoonotic Dis 18(10): 519-523.
236. Torén, K., et al. (2020). "Occupational exposure to dust and to fumes, work as a welder and invasive pneumococcal disease risk." OCCUP ENVIRON MED 77(2): 57-63.
237. Torres-Gonzalez, P., et al. (2013). "Prevalence of latent and active tuberculosis among dairy farm workers exposed to cattle infected by Mycobacterium bovis." PLoS Negl Trop Dis 7(4): e2177.

238. Toseva, E. I., et al. (2018). "Seroprevalence of anti-HAV total antibodies among workers in wastewater treatment plants." Int J Occup Med Environ Health 31(3): 307-315.
239. Traoré, K. A., et al. (2015). "Hepatitis E virus exposure is increased in pork butchers from Burkina Faso." AM J TROP MED HYG 93(6): 1356-1359.
240. Tschopp, A., et al. (2009). "Hepatitis E, Helicobacter pylori and peptic ulcers in workers exposed to sewage: A prospective cohort study." OCCUP ENVIRON MED 66(1): 45-50.
241. Vainio, A., et al. (2009). "An outbreak of pneumonia associated with S. pneumoniae at a military training facility in Finland in 2006." APMIS 117(7): 488-491.
242. van Cleef, B. A. G. L., et al. (2010). "High prevalence of nasal MRSA carriage in slaughterhouse workers in contact with live pigs in the Netherlands." EPIDEMIOL INFECT 138(5): 756-763.
243. van Cleef, B. A. G. L., et al. (2011). "Persistence of livestock-associated methicillin-resistant Staphylococcus aureus in field workers after short-term occupational exposure to pigs and veal calves." J CLIN MICROBIOL 49(3): 1030-1033.
244. van Veen, M. G., et al. (2010). "HIV and sexual risk behavior among commercial sex workers in the Netherlands." Arch Sex Behav 39(3): 714-723.
245. Verhaegh-Haasnoot, A., et al. (2015). "High burden of STI and HIV in male sex workers working as internet escorts for men in an observational study: a hidden key population compared with female sex workers and other men who have sex with men." BMC Infect Dis 15: 291.
246. Vilay, P., et al. (2019). "Malaria prevalence, knowledge, perception, preventive and treatment behavior among military in Champasak and Attapeu provinces, Lao PDR: A mixed methods study." Trop Med Health 47(1).
247. Vorsters, A., et al. (2016). "Prevalence of high-risk human papillomavirus and abnormal pap smears in female sex workers compared to the general population in Antwerp, Belgium." BMC Public Health 16: 477.
248. Vu, L. and K. Misra (2018). "High Burden of HIV, Syphilis and HSV-2 and Factors Associated with HIV Infection Among Female Sex Workers in Tanzania: Implications for Early Treatment of HIV and Pre-exposure Prophylaxis (PrEP)." AIDS Behav 22(4): 1113-1121.
249. Wadl, M., et al. (2010). "Food-borne norovirus-outbreak at a military base, Germany, 2009." BMC Infect Dis 10: 30.
250. Wang, X., et al. (2014). "Seroprevalence to avian influenza A(H7N9) virus among poultry workers and the general population in Southern China: A longitudinal study." CLIN INFECT DIS 59(6): e76-83.
251. Wang, X., et al. (2013). "Hospital-based prevalence of high-risk cervical HPV types infecting the general population and female sex workers in Huzhou, China." Int J Gynecol Obstet 120(1): 37-41.
252. Watier-Grillot, S., et al. (2017). "Challenging Investigation of a Norovirus Foodborne Disease Outbreak During a Military Deployment in Central African Republic." Food Environ Virol 9(4): 498-501.
253. Wickersham, J. A., et al. (2017). "Prevalence of Human Immunodeficiency Virus and Sexually Transmitted Infections among Cisgender and Transgender Women Sex Workers in Greater Kuala Lumpur, Malaysia: Results from a Respondent-Driven Sampling Study." SEX TRANSM DIS 44(11): 663-670.

254. Wilczynski, J. A., et al. (2012). "Outbreak of cryptosporidiosis associated with a firefighting response - Indiana and Michigan, June 2011." Morb Mortal Wkly Rep 61(9): 153-156.
255. Wilken, J. A., et al. (2014). "Coccidioidomycosis among cast and crew members at an outdoor television filming event--California, 2012." MMWR Morb Mortal Wkly Rep 63(15): 321-324.
256. Williams, C. J., et al. (2013). "Risk exposures for human ornithosis in a poultry processing plant modified by use of personal protective equipment: an analytical outbreak study." EPIDEMIOL INFECT 141(9): 1965-1974.
257. Wilson, L. E., et al. (2010). "Investigation of a Q fever outbreak in a Scottish co-located slaughterhouse and cutting plant." Zoonoses Public Health 57(7-8): 493-498.
258. Wongsanuphat, S., et al. (2020). "Investigation of measles outbreak among thai and migrant workers in two factories in Nakhon Pathom, Thailand, 2019." Int J Environ Res Public Health 17(13): 1-11.
259. Wu, J., et al. (2015). "Anti-Human H1N1pdm09 and swine H1N1 Virus Antibodies among Swine Workers in Guangdong Province, China." Sci Rep 5: 12507.
260. Wu, J. Y., et al. (2019). "Transmission risk of avian influenza virus along poultry supply chains in Guangdong, China." J INFECT 79(1): 43-48.
261. Yang, S., et al. (2014). "Avian-origin influenza A(H7N9) infection in influenza A(H7N9)-affected areas of China: a serological study." J INFECT DIS 209(2): 265-269.
262. Ye, X., et al. (2016). "Livestock-associated methicillin and multidrug resistant S. aureus in humans is associated with occupational pig contact, not pet contact." Sci. rep. 6: 19184.
263. Ye, X., et al. (2015). "Frequency-risk and duration-risk relations between occupational livestock contact and methicillin-resistant Staphylococcus aureus carriage among workers in Guangdong, China." AM J INFECT CONTROL 43(7): 676-681.
264. Yoon, C. G., et al. (2019). "The infectivity of pulmonary tuberculosis in Korean army units: Evidence from outbreak investigations." Tuberc Respir Dis 82(4): 298-305.
265. Younis, F., et al. (2020). "Respiratory health disorders associated with occupational exposure to bioaerosols among workers in poultry breeding farms." Environ Sci Pollut Res Int. 10.1007/s11356-020-08485-x.
266. Yu, J., et al. (2020). "Vietnamese female sex workers in rural cross-border areas of Guangxi, China: migration and HIV/STI risk behaviors." Aids Care Feb 3;1-9.
267. Yu, P., et al. (2013). "Outbreak of acute respiratory disease caused by human adenovirus type 7 in a military training camp in Shaanxi, China." MICROBIOL IMMUNOL 57(8): 553-560.
268. Zakutna, L., et al. (2015). "Sero-epidemiological study of Lyme disease among high-risk population groups in eastern Slovakia." Ann Agric Environ Med 22(4): 632-636.
269. Zalla, L. C., et al. (2019). "The burden of HIV among female sex workers, men who have sex with men and transgender women in Haiti: results from the 2016 Priorities for Local AIDS Control Efforts (PLACE) study." J Int AIDS Soc 22(7): e25281.
270. Zermiani, M., et al. (2012). "Prevalence of sexually transmitted diseases and Hepatitis C in a survey of female sex workers in the North-east of Italy." Open AIDS J 6(1): 60-64.
